# Identification and Distribution of Potentially Azole‐Resistant Airborne Fungi in Outdoor Environments of the Basque Country

**DOI:** 10.1111/1758-2229.70152

**Published:** 2025-10-29

**Authors:** Saioa Cendon‐Sanchez, Eduardo Pelegri‐Martinez, Uxue Perez‐Cuesta, Xabier Guruceaga, Andoni Ramirez‐Garcia, Ana Abad‐Diaz‐de‐Cerio, Aitor Rementeria

**Affiliations:** ^1^ Department of Immunology, Microbiology and Parasitology University of the Basque Country (UPV/EHU) Leioa Bizkaia Spain; ^2^ Department of Clinical Pharmacy and Translational Science Pharmacy College, University of Tennessee Health Science Center (UTHSC) Memphis Tennessee USA; ^3^ Institute for Multidisciplinary Research in Applied Biology (IMAB), Public University of Navarre (UPNA) Pamplona Spain

**Keywords:** airborne fungi, azole resistance, environmental samplings, fungal diversity, outdoor environment, rural and urban areas

## Abstract

Airborne fungi are ubiquitous microorganisms in the environment, and some of them are known opportunistic pathogens. In recent years, azole resistance, which can have a clinical or environmental origin, has become a critical issue. Four environmental samplings were performed to assess the prevalence and diversity of potentially azole‐resistant fungi in three areas (hospital surrounding, rural and urban) from the Basque Country. The microbial concentration varied from 40 to 3670 CFU/m^3^, depending on the location and sampling. The CFU/m^3^ count on plates incubated with voriconazole at 37°C was only three times lower than that of plates incubated without the antifungal, suggesting many 37°C‐growing fungi might be triazole‐resistant. Three hundred and twenty one potentially resistant isolates were identified, belonging to 21 genera and 55 species. *Alternaria* (62.31%) and *Talaromyces* (18.69%) were the predominant genera, with *Alternaria infectoria* (47.19%) being the most abundant species. Overall, the two coastal provinces (Bizkaia and Gipuzkoa) showed the most similarity. The rural area exhibited the highest alpha diversity values for each province, whereas samples from this area were more alike in terms of beta diversity. PCoA analysis indicated that sampling points were clustered by samplings or provinces. This study provides the first characterisation of the outdoor environment from the Basque Country and highlights the importance of determining the prevalence of potentially azole‐resistant isolates.

## Graphical abstract

Air samples collected at three areas in each province of the Basque Country (Spain) were incubated at 25°C and 37°C for Sabouraud plates (SAB) and at 37°C for plates supplemented with voriconazol (SAB‐VCZ). The potentially resistant isolates from SAB‐VCZ plates were identified and their abundance and diversity were determined.

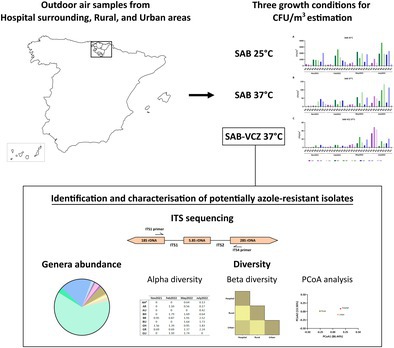

## 1 Introduction

Fungi constitute a ubiquitous group of microorganisms that are present naturally in the environment and play a crucial role in ecological processes; for instance, they are involved in the decomposition and recycling of organic matter (Anees‐Hill et al. 2022; Cvetnic and Pepeljnjak 1997). As they can be dispersed through air, fungal spores constitute a considerable proportion of the biological aerosol particles (Martinez‐Bracero et al. 2022). Many studies have focused on identifying airborne fungi from outdoor environments of different countries (Adhikari et al. 2004; Kasprzyk and Worek 2006; Nageen et al. 2023; Oliveira et al. 2010; Shelton et al. 2002). In Spain, the presence of these fungi has been studied in different regions such as the south of the country (Elvira‐Rendueles et al. 2013; Fernández‐Rodríguez et al. 2014), Madrid (Herrero et al. 2006; Sabariego et al. 2007) and Catalonia (Vélez‐Pereira et al. 2016). However, in other regions such as the Basque Country, no outdoor environmental samplings have been carried out to detect airborne fungi. In addition to the geographical location, fungal concentration varies depending on seasonality, which is determined by factors such as meteorological conditions, vegetation and human activities (Anees‐Hill et al. 2022; Grinn‐Gofroń et al. 2020).

The environmental sampling studies have shown that species of *Cladosporium*, *Alternaria*, *Penicillium* and *Aspergillus* genera are the most common fungi in outdoor environments (Adhikari et al. 2004; Fernández‐Rodríguez et al. 2014; Nageen et al. 2023; Shelton et al. 2002; Soto et al. 2009). These taxa include environmental phytopathogenic fungi, which are responsible for approximately 20% of perennial crop losses worldwide (Fisher et al. 2018), but also fungi known to cause allergic responses (Fukutomi and Taniguchi 2015) and opportunistic infections, such as those triggered by *Aspergillus fumigatus* (Ferrer and Alió 2011; Latgé and Chamilos 2019; Lo Porto et al. 2023; Sandoval‐Denis et al. 2015).

The emergence of antifungal resistance represents a major global concern, particularly with regard to triazoles. Clinical triazoles, which include voriconazole (VCZ), itraconazole and posaconazole, among others, constitute one of the most frequently administered groups of antifungal compounds for prevention or treatment of fungal infections (Agarwal et al. 2024; Patterson et al. 2016; Walsh et al. 2008). Prolonged treatment in chronic patients or the use of azoles, such as difenoconazole and tebuconazole, in agriculture (Toda et al. 2021) can lead to resistance development (Howard et al. 2009; Snelders et al. 2009; Tashiro et al. 2012). The similar structure and mechanism of action of both agricultural and clinical azoles can additionally contribute to the emergence of cross‐resistance in opportunistic fungi (Snelders et al. 2012). It is noteworthy that azoles are the most widely used antifungals to control plant diseases in the EU (Fisher et al. 2018). Thus, some studies have been carried out in different countries to evaluate the resistance to azoles of environmental fungal species, mainly *A. fumigatus* , with different resistance rates ranging from 3%–10% (Arendrup et al. 2024; Chen et al. 2020; Chowdhary, Kathuria, et al. 2012; Tangwattanachuleeporn et al. 2017; Tsitsopoulou et al. 2018). In Spain, there are only a few studies investigating the antifungal prevalence in *Aspergillus* species (Álvarez‐Pérez et al. 2023; Mortensen et al. 2010).

Therefore, given the absence of current data on the composition of environmental mycobiota and their associated antifungal resistances in our community, in this study we aim to describe the airborne fungal diversity and distribution of potentially azole‐resistant isolates from the Basque Country, an Autonomous Community located in the north of Spain, through different seasons. In order to obtain detailed information, we collected air from the three provinces that constitute the Basque Country, including in each location three outdoor sampling points: a hospital surrounding, a rural area and an urban area.

## 2 Experimental Procedures

### 2.1 Sampling Locations

**FIGURE 1:**
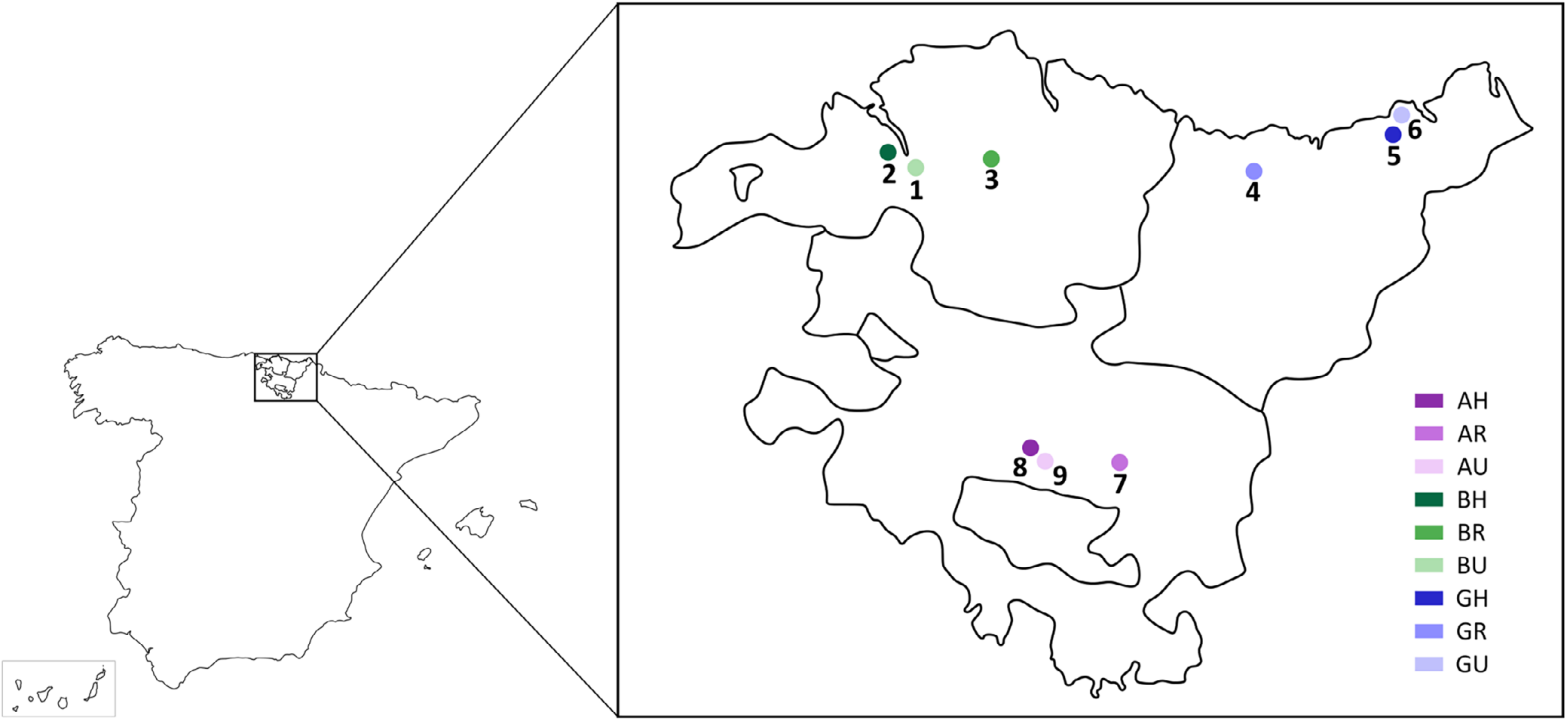
Map of the Basque Country indicating the nine sampling points. The numbered points represent the sampling locations of each province in the same order in which the route was completed. Each sampling point is shown in different shades of colour: purple (Araba), green (Bizkaia) or blue (Gipuzkoa). AH, Araba Hospital; AR, Araba Rural; AU, Araba Urban; BH, Bizkaia Hospital; BR, Bizkaia Rural; BU, Bizkaia Urban; GH, Gipuzkoa Hospital; GR, Gipuzkoa Rural; GU, Gipuzkoa Urban.

The sampling locations selected for this study belonged to the Autonomous Community of the Basque Country, which consists of three different provinces: Araba, Bizkaia and Gipuzkoa (Figure 1). This Autonomous Community is located in the north of Spain and has a population of about 2.2 million inhabitants. These provinces show variable density: Araba is the least populated (109.85 inhabitants/km^2^), followed by Gipuzkoa and Bizkaia with 360.48 and 516.02 inhabitants/km^2^, respectively (EUSTAT 2024). Bizkaia and Gipuzkoa are coastal locations, whereas Araba is inland. The entire region exhibits a heterogeneous climate (Euskalmet n.d). Bizkaia, Gipuzkoa and the north of Araba present an Atlantic climate, characterised by moderate temperatures (Ts), humidity and rain. On the other hand, the majority of Araba exhibits a combination of subatlantic and submediterranean climate, with fewer precipitations and warmer summers.

Outdoor air samplings were conducted at three sampling points in each province that were divided by area: hospital surrounding, rural and urban. In Araba, the locations were Txagorritxu Hospital (AH; 42°51′14.2″ N 2°41′32.2″ W), Iruraiz‐Gauna (AR; 42°51′29.2″ N 2°29′35.2″ W) and Plaza de los Fueros (AU; 42°50′44.2″ N 2°40′12.4″ W). In Bizkaia, Cruces University Hospital (BH; 43°16′56.4″ N 2°59′00.5″ W), Larrabetzu (BR; 43°16′01.8″ N 2°48′25.8″ W) and Plaza Indautxu (BU; 43°15′38.3″ N 2°56′25.9″ W). In Gipuzkoa, Donostia University Hospital (GH; 43°17′28.6″ N 1°58′26.2″ W), Arroa Goikoa (GR; 43°15′14.6″ N 2°16′23.6″ W) and Plaza Cataluña (GU; 43°19′25.3″ N 1°58′28.8″ W).

### 2.2 Air Samplings and Incubation Conditions

Four environmental samplings were performed between 2021 and 2022, one in each season: November 2021 (Nov2021), February 2022 (Feb2022), May 2022 (May2022) and July 2022 (July2022). Air samples were collected using the MAS‐100 Eco Air Sampler (MBV AG, Stäfa, Switzerland), which operated at a 100 L/min flow rate. The sampler was disinfected with 70% ethanol between each sampling point to prevent cross‐contamination. For every condition, a minimum volume of 50 L impacted directly onto Sabouraud agar plates (SAB; Condalab, Madrid, Spain) or SAB plates supplemented with 1 mg/L of VCZ (Acros Organics, Geel, Belgium) (SAB‐VCZ). The air sampler operated for 1, 2 and 10 min for SAB plates incubated at 25°C and 37°C and SAB‐VCZ plates, respectively.

All air samples were collected on the same day following the same route every time to avoid variations within each sampling point. Environmental factors data, both T and humidity, were obtained from Agencia Estatal de Meteorología from Spain (Aemet; https://www.aemet.es/es/) for each location at the sampling timepoint. The four samplings were conducted on a sunny day, with no rain registered the previous day.

SAB plates were incubated at both 25°C and 37°C, as environmental T or human T, respectively. SAB‐VCZ plates were incubated only at 37°C to detect potential human fungal pathogens. All plates were observed every 24 h to assess colony growth. After 72 h of incubation, all colony forming units (CFU) were counted. Then, colonies from SAB‐VCZ plates were aseptically isolated and subcultured onto fresh potato‐dextrose agar plates (PDA; Condalab) for later fungal identification. These isolates are referred to as potentially antifungal‐resistant, as no standardised methods have been performed to confirm their resistance. Their abundance is referred throughout the document to the number of isolates.

### 2.3 Fungal Identification

After 48–72 h of incubation, depending on the fungus growth rate, on fresh PDA at 37°C, DNA extraction of the colonies was carried out as previously described (Hervás‐Aguilar et al. 2007) with minor modifications in the following steps: Tubes were incubated at 65°C (instead of 70°C) for 30 min and 100 μL of phenol/chloroform/isoamyl alcohol (25:24:1) was added (instead of 200 μL). Primers ITS1 (TCCGTAGGTGAACCTGCGG) and ITS4 (TCCTCCGCTTATTGATATGC) were used for the amplification of the extracted DNA by PCR (White et al. 1990). PCR mix was composed of 12.5 μL DreamTaq PCR Master mix (1×) (Thermo Fisher Scientific, Waltham, MA, USA), 1 μL of 1:10 diluted DNA, 0.2 μL of BSA (10 mg/mL), and 1 μL of each primer (10 μM); the remaining volume up to 25 μL consisted of PCR‐grade water (Thermo Fisher Scientific). Amplification conditions used were: an initial denaturation step at 95°C for 3 min, followed by 35 cycles of amplification steps (denaturation at 95°C for 30 s, annealing at 56°C for 30 s and extension at 72°C for 90 s), and a final extension step at 72°C for 10 min. MJ‐Mini thermal cycler (Bio‐Rad, Hercules, CA, USA) was used. Amplicons were analysed by 1% agarose gel electrophoresis in 1× TAE buffer (40 mM Tris‐acetate and 1 mM EDTA) stained with GelRed (Biotium, Fremont, CA, USA) at 90 V for 45 min. The gel was visualised using a U:GENIUS transilluminator (Syngene, Bangalore, India).

The resulting products were purified using the ADN NZY Gelpure kit (NZYtech, Lisboa, Portugal) following the manufacturer's protocol, and quality was assessed with the Nanodrop lite spectrophotometer (Thermo Fisher Scientific). Samples were sequenced at Eurofins Genomics (Ebersberg, Germany) and the isolates were identified using Basic Local Alignment Search Tool (BLAST, https://blast.ncbi.nlm.nih.gov/Blast.cgi) with default settings.

### 2.4 Statistical Analysis

Data normality distribution was assessed using the Shapiro–Wilk test (*p* < 0.05 was considered not normal distribution) and all samples were found to be non‐parametric. The Kruskal–Wallis test was performed to detect variations in CFU/m^3^ as well as in genera abundance. Post hoc Dunn's tests were used for pairwise comparison. The effect of environmental parameters was also evaluated using Spearman correlation (*p* < 0.05 was considered significant). Statistical analysis was performed using IBM SPSS Statistics (v.28.0.1.1).

To analyse fungal diversity, Shannon index and Bray–Curtis dissimilarities were used for alpha and beta diversity, respectively. Principal co‐ordinates analysis (PCoA) and the diversity analysis were performed using the vegan package (v.2.6.4) in R. Graphs were made using GraphPad Prism v.8.0.2. Venn diagrams package (v.1.7.3) in R was used to plot data and to determine the exclusivity of the fungal species of the two most abundant genera within each aggrupation.

## 3 Results

### 3.1 Microbial Concentration in the Basque Country Provinces

**FIGURE 2:**
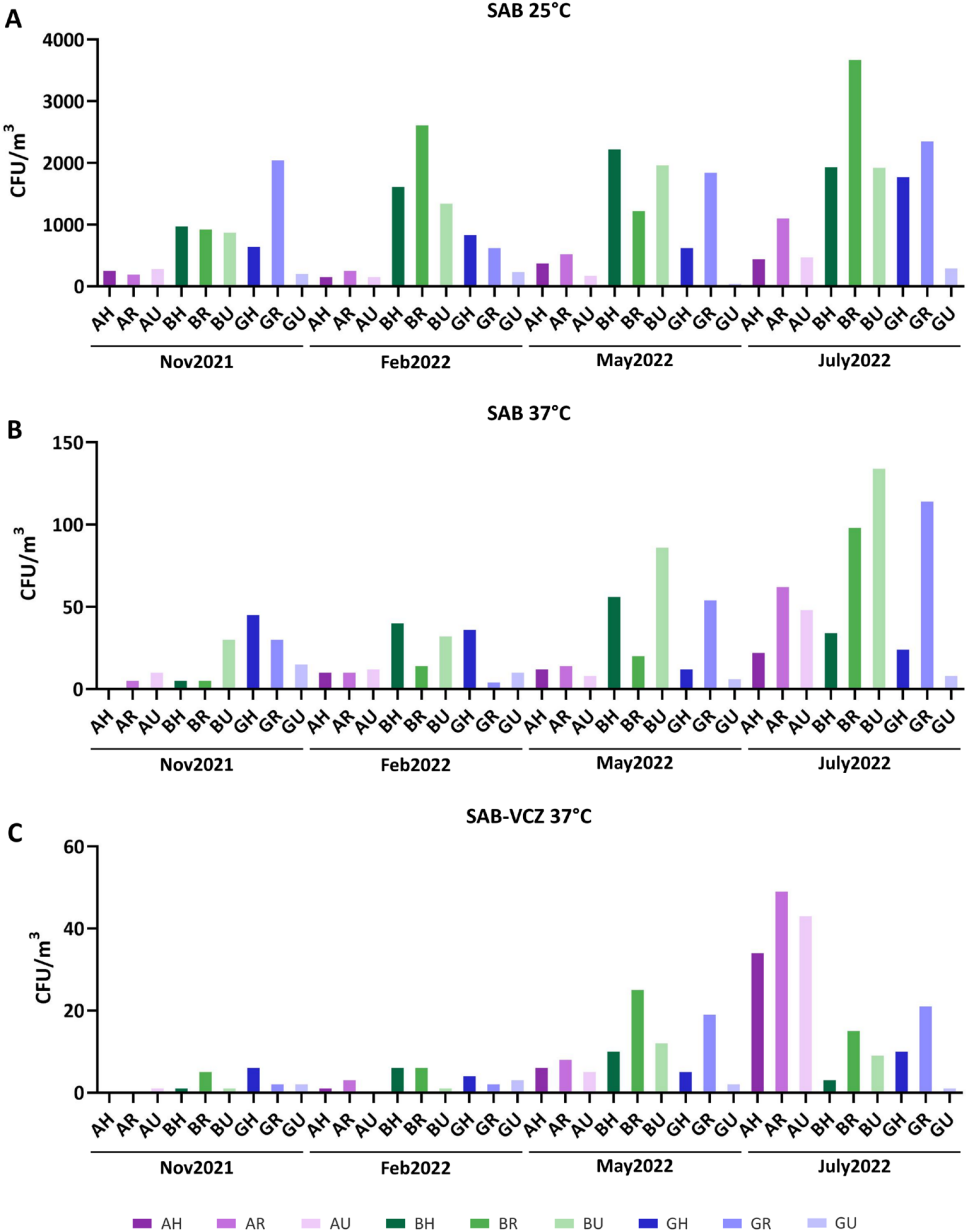
Total counts of colony forming units per volume of filtered air (CFU/m^3^). Three different growth conditions are shown: SAB—Sabouraud plates incubated at 25°C (A) and at 37°C (B) and SAB‐VCZ—Sabouraud plates supplemented with 1 mg/L of voriconazole incubated at 37°C (C). Each sampling point is shown in different shades of colour: purple (Araba), green (Bizkaia) or blue (Gipuzkoa). AH: Araba Hospital. AH, Araba Hospital; AR, Araba Rural; AU, Araba Urban; BH, Bizkaia Hospital; BR, Bizkaia Rural; BU, Bizkaia Urban; GH, Gipuzkoa Hospital; GR, Gipuzkoa Rural; GU, Gipuzkoa Urban.

In the four performed samplings, SAB plates were incubated at two Ts: 25°C (Figure 2A), representing environmental T, and 37°C (Figure 2B), human pathogens T. SAB‐VCZ plates were incubated exclusively at 37°C (Figure 2C). The total number of CFUs varied among incubation Ts; however, the trends were similar regarding samplings and provinces. As expected, the increase of incubation T acted as an important restriction factor, with reductions of more than 30‐fold in the CFU/m^3^ observed (Figure 2B). Likewise, the presence of the antifungal in SAB‐VCZ plates affected fungal growth, but to a much lesser extent, the number of CFU/m^3^ decreased to only one‐third in almost all samples (Figure 2C). Additionally, as observed in Figure 2, samples from summer, July2022, showed, on average, the highest CFU/m^3^, being the mean values around 1600 CFU/m^3^ at 25°C, 60 CFU/m^3^ at 37°C on SAB and 20 CFU/m^3^ on SAB‐VCZ plates. Between provinces, Bizkaia samples showed more elevated counts in almost every location. Finally, samples from rural areas showed higher mean values than samples from hospital and urban areas for the environmental condition (Nov2021: 620, 1050 and 450 CFU/m^3^; Feb2022: 863.33, 1160 and 573.33 CFU/m^3^, May2022: 1170, 1193.33 and 723.33 CFU/m^3^, July2022: 1380, 2373.33 and 893.33 CFU/m^3^; for hospital, rural and urban areas, respectively) (Figure S1). However, in general, there were no significant differences.

Euskalmet (Basque meteorological agency) reports showed that November was very humid and rainy, with cold mean Ts, between 10°C and 11°C in the coast and 7°C in Araba (Euskalmet 2021). February was a warmer month, with a 1.1°C increase based on 1981–2010 period mean values and showed less precipitation (Euskalmet 2022a). Then, May was a month with warm Ts and was reported as one of the driest Mays compared to a 20‐year period from 1981 to 2010 (Euskalmet 2022b). Finally, July was similar to May, but with higher mean Ts, around 21°C in all provinces (Euskalmet 2022c). Spearman correlation was used to analyse the effect of environmental factors, T and relative humidity (RH), regarding the data collected on each sampling day (Table S1). Overall, CFU/m^3^ values were positively correlated with T (values range from 0 to 1) and negatively with RH (values range from −0.32 to −1), this being more evident in SAB plates with no antifungal (Table S2). Significant correlations (*p* < 0.05) between CFU/m^3^ and both environmental factors (*ρ* = 1.0 with T and *ρ* = −1.0 with RH) were only reported in samples from Araba (AH and AR). This significant effect regarding T was only detected in another sample, GR, incubated without antifungal at 37°C (*ρ* = 1.0).

### 3.2 Potentially Azole‐Resistant Isolates Identification, Diversity and Area Distribution

**FIGURE 3:**
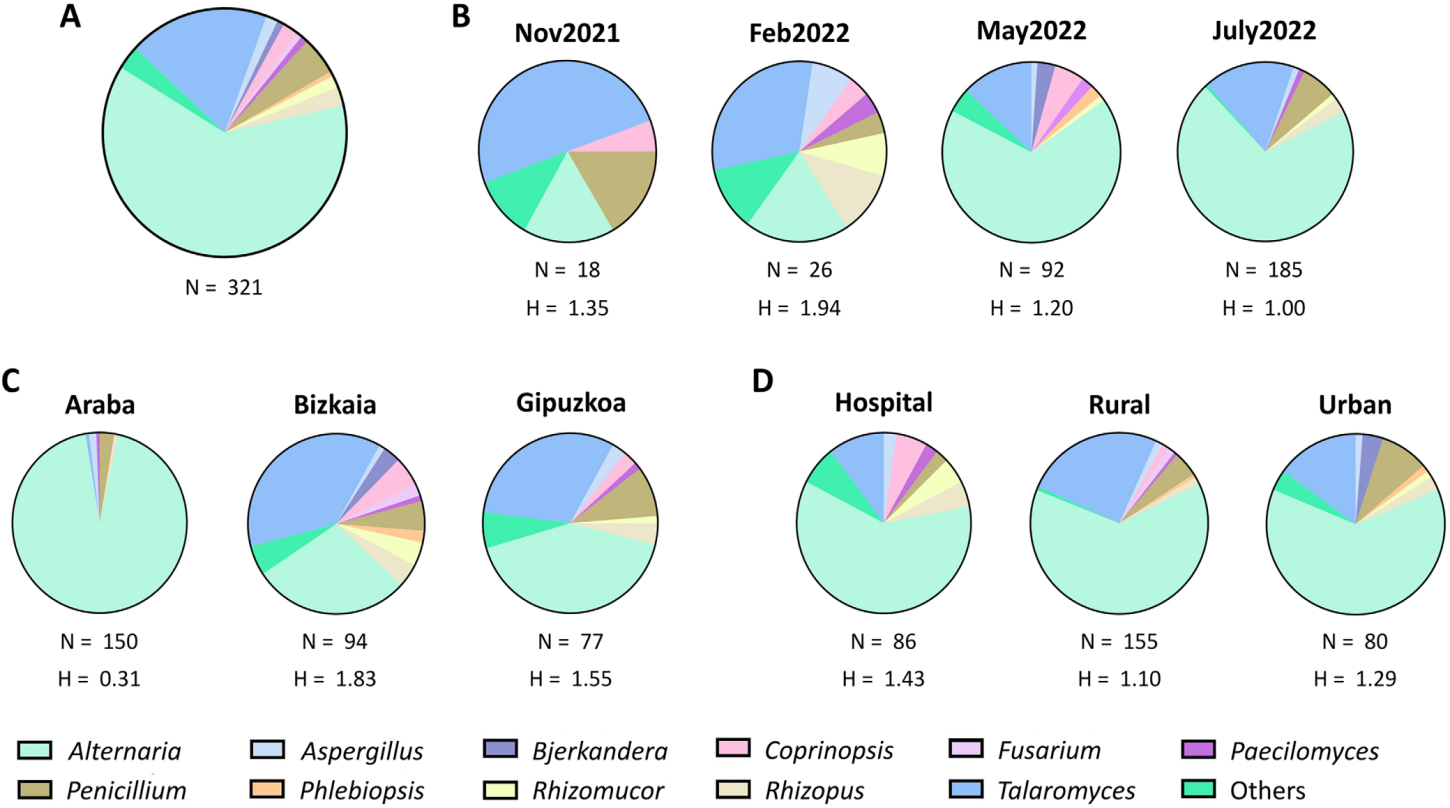
Relative abundance of the most dominant genera isolated from SAB‐VCZ plates. Different representations of the same data are shown: general distribution (A), by sampling (B), by province (C) and by area (D). The number of isolates (*N*) and the Shannon index values (*H*) are indicated below each representation. Others include the genera that are represented less than 0.5% among the total of the isolated colonies. Each genera is shown in a colour.

To assess the prevalence of potentially resistant species to VCZ, all colonies that grew on SAB‐VCZ were isolated and identified. ITS sequencing enabled identification of 321 isolates belonging to 21 genera and 55 species (Figure 3A and Table S3). Only seven species identifications had an identity percent below 97%, and just one colony was identified exclusively at the genus level, which highlights the reliability of the results. Despite high‐quality sequencing results, we were not able to discriminate between two or more species for some isolates (9.4%), as both score and hits from BLAST were similar. As noted above, more isolates (*n* = 185) were recorded from summer samples (July2022), followed by samples from spring (May2022), winter (Feb2022) and autumn (Nov2021) with 92, 26 and 18 isolates, respectively (Figure 3B). These isolates (*n* = 321) were distributed among different provinces or areas as follows: 150, 94 and 77 isolates from samples from Araba, Bizkaia and Gipuzkoa provinces, respectively (Figure 3C); or 86, 155 and 80 isolates from samples from hospital surrounding, rural and urban areas, respectively (Figure 3D).

**FIGURE 4:**
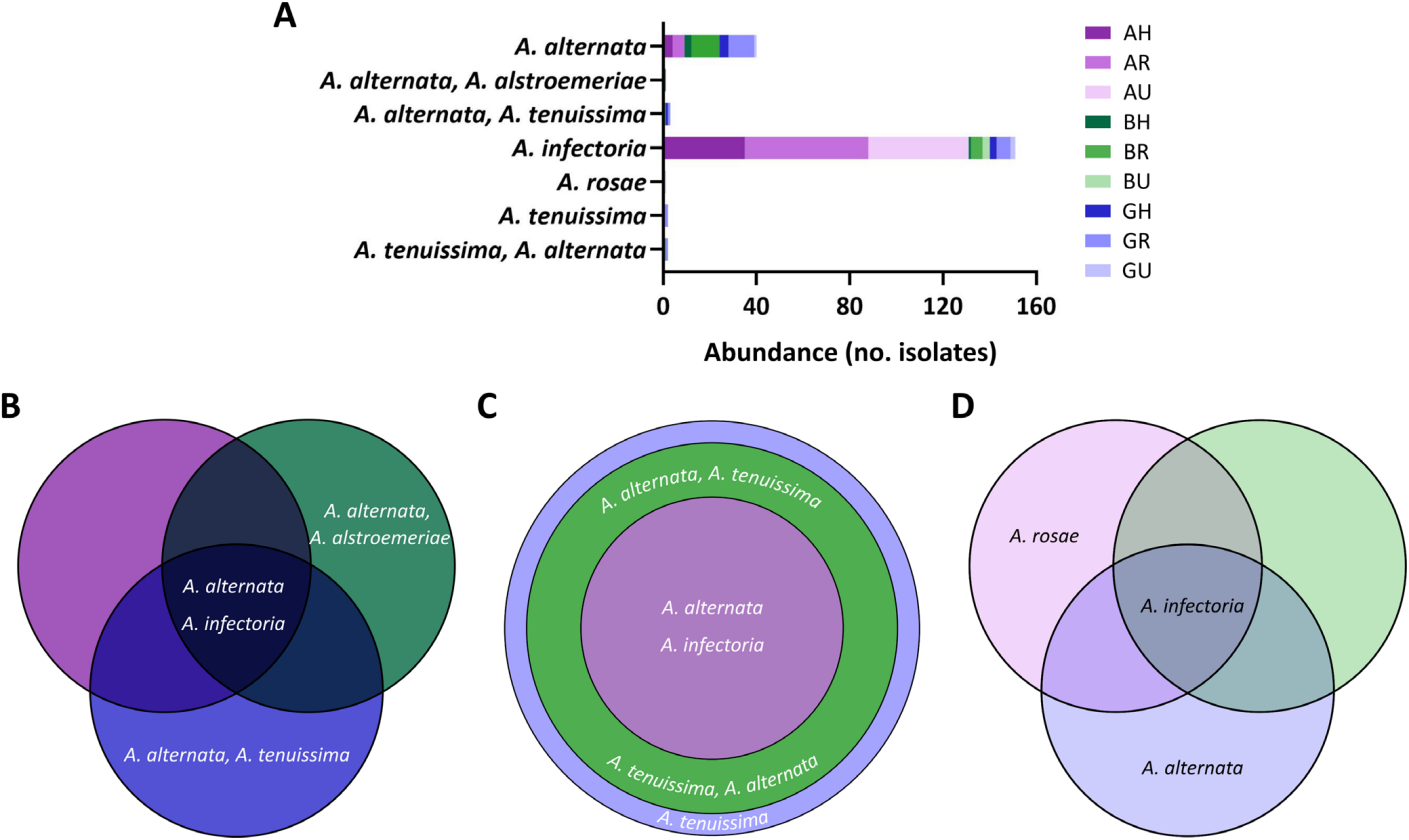
*Alternaria* spp. representation in the nine sampling points. Abundance in counts of the *Alternaria* spp. (A). Venn diagrams show the distribution of the species in hospital surrounding (B), rural (C) and urban areas (D). Each sampling point is shown in different shades of colour: purple (Araba), green (Bizkaia) or blue (Gipuzkoa). AH, Araba Hospital; AR, Araba Rural; AU, Araba Urban; BH, Bizkaia Hospital; BR, Bizkaia Rural; BU, Bizkaia Urban; GH, Gipuzkoa Hospital; GR, Gipuzkoa Rural; GU, Gipuzkoa Urban.

Overall, species of *Alternaria* and *Talaromyces* genera were the most detected between potentially resistant isolates (Figure 3). *Alternaria* was the most abundant genus, accounting for more than 60% of the isolates (Figure 3A), and it represented more than 65% of the identified isolates in samples of May2022 (67.39%) and July2022 (70.27%) (Figure 3B). Identified *Alternaria* species were few diverse (Figure 4A). In fact, the most abundant species was *Alternaria infectoria* , which represented 75.5% of the total; it was detected in every sampling point (Figure 4B–D) and was more common in July2022 samples (62.5%). *Alternaria alternata* was the second most isolated (20%; 40 isolates); nevertheless, sometimes the BLAST database showed that results were similar for both *Alternaria alstroemeriae* and *Alternaria tenuissima* (13%; 6 isolates out of 46). Besides, except in Gipuzkoa samples, *A. alternata* was exclusively detected in samples from hospital surrounding and rural areas (Figure S2).

**FIGURE 5:**
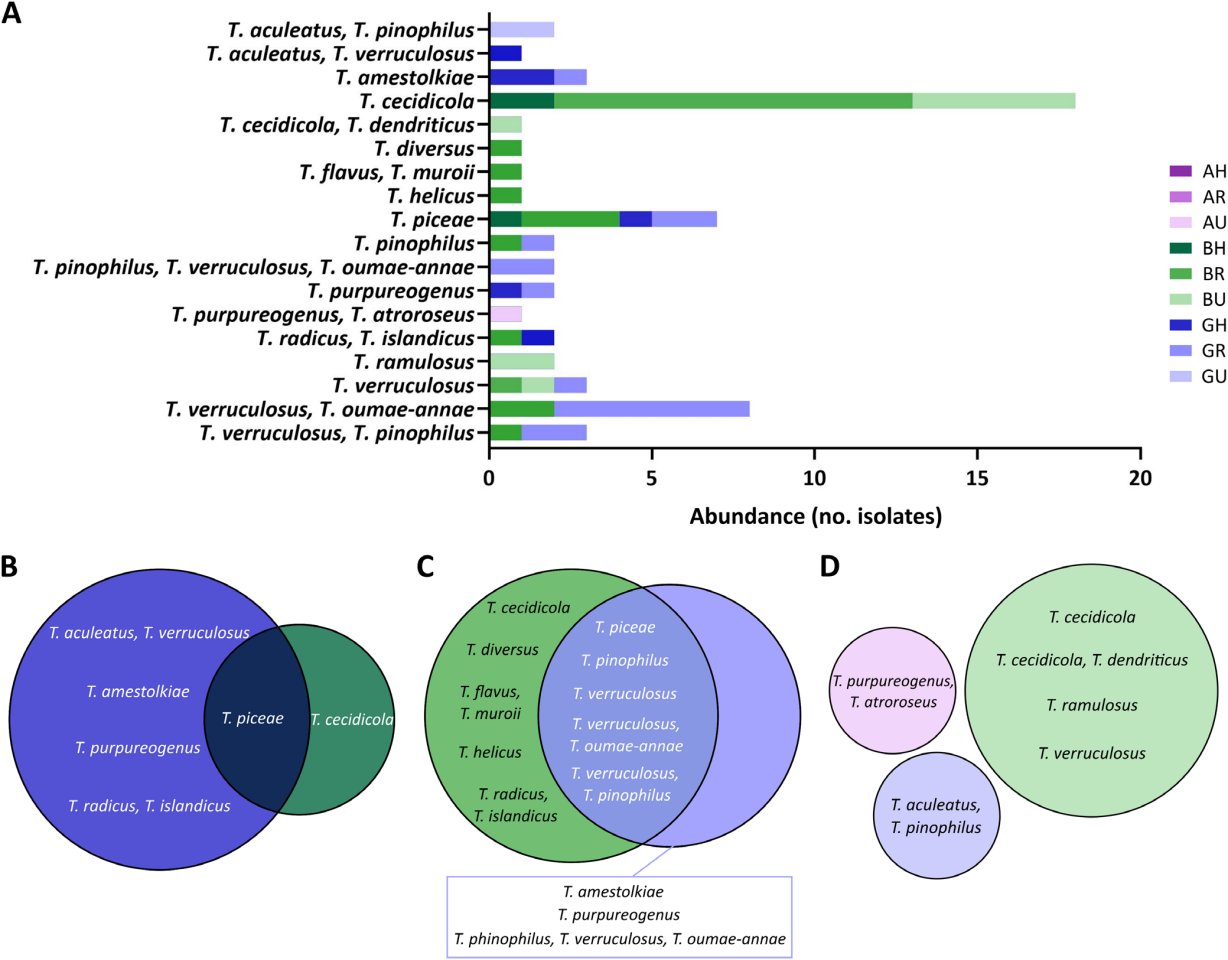
*Talaromyces* spp. representation in the nine sampling points. Abundance in counts of the *Talaromyces* spp. (A). Venn diagrams show the distribution of the species in hospital surrounding (B), rural (C) and urban areas (D). Each sampling point is shown in different shades of colour: purple (Araba), green (Bizkaia) or blue (Gipuzkoa). AH, Araba Hospital; AR, Araba Rural; AU, Araba Urban; BH, Bizkaia Hospital; BR, Bizkaia Rural; BU, Bizkaia Urban; GH, Gipuzkoa Hospital; GR, Gipuzkoa Rural; GU, Gipuzkoa Urban.

*Talaromyces* was also identified in a substantial proportion (18.69%) (Figure 3A) showing the highest species richness; 18 *Talaromyces* species were detected (Figure 5A). Overall, more isolates of this genus were identified by BLAST with two or more species compared to *Alternaria* isolates. Besides, it was the most dominant genus of samples from Nov2021 and Feb2022, representing 50% and 30.77% of the isolates, respectively (Figure 3B). Bizkaia was the region with more *Talaromyces* isolates, 58.33% compared with 40% in Gipuzkoa and 1.67% in Araba (Figure 3C). *Talaromyces cecidicola* was the dominant species (30%), which was exclusive to Bizkaia samples (Figure 5B–D) and it was the only common species between the three sampling points of this province (Figure S3A). In hospital area samples, *Talaromyces piceae* was the only species in common between samples from Bizkaia and Gipuzkoa (Figure 5B), whereas in rural areas, another four species of this genus were shared by samples of these provinces (Figure 5C). Interestingly, no species were shared in urban areas, but it was the only location where *Talaromyces* species were identified in Araba (Figure 5D). Moreover, the isolates identified as *Talaromyces aculeatus,* *Talaromyces pinophilus* were exclusive to Gipuzkoa urban samples, which had no shared species with other samples from the province (Figure S3B).

The remaining genera represented a much lower percentage of the isolates, with *Penicillium* spp. reaching a maximum of 5%, *Rhizopus* spp. (2.49%), *Coprinopsis* spp. (2.18%), *Aspergillus* spp. (1.56%), *Rhizomucor* spp. (1.56%), *Bjerkandera* spp. (0.93%), *Paecilomyces* spp. (0.93%), *Fusarium* spp. (0.62%) and *Phlebiopsis* spp. (0.62%) (Figure 3A). All species that belonged to less prevalent genera were included in ‘Others’ (3.11%), which groups together species of genera such as *Cephalotrichum*, *Coprinellus*, *Curvularia*, *Hyphodermella*, *Lichtheimia*, *Microascus*, *Phanerochaete*, *Polyporus*, *Rasamsonia* and *Syncephalastrum*. In addition, species of *Aspergillus*, *Paecilomyces*, *Penicillium* and *Rhizopus* were also common across the three provinces. Finally, it should be noted that some of the identified potentially antifungal‐resistant species belonged to important opportunistic pathogens. Most of them corresponded to the following well‐known genera: *Alternaria*, *Aspergillus*, *Rhizomucor* and *Rhizopus*, but also to rare opportunistic genera such as *Lichtheimia* or *Syncephalastrum*.

As for microbial concentration, the effect of environmental factors, T and RH (Table S1), on the distribution of the identified genera was assessed using Spearman correlation, but no clear trend was detected in this case (data not shown). Regarding diversity, based on genera abundance, lower alpha diversity was reported for the warmer months, which could be correlated with the higher prevalence of *Alternaria* and *Talaromyces* in samples from spring and summer (Figure 3B). Bray–Curtis Dissimilarity results indicated that the samplings from the colder months were not similar to the warmer ones (Figure S4A). However, PCoA analysis only showed a close relation between Nov2021 and Feb2022 (Figure S4B). Overall, Araba was the province showing the lowest alpha diversity of potentially antifungal‐resistant fungi (Figure 3C), due to the high number of isolates corresponding to *Alternaria* species (94%). Besides, it was the most discrepant region as observed for beta diversity (Figure S4C) and PCoA analysis (Figure S4D), where both coastal regions showed great similarity. All areas revealed uniformity in the alpha (Figure 3D) and beta diversity (Figure S4E) results; however, the two urban locations (urban and hospital areas) seemed to be closely related (Figure S4F).

**FIGURE 6:**
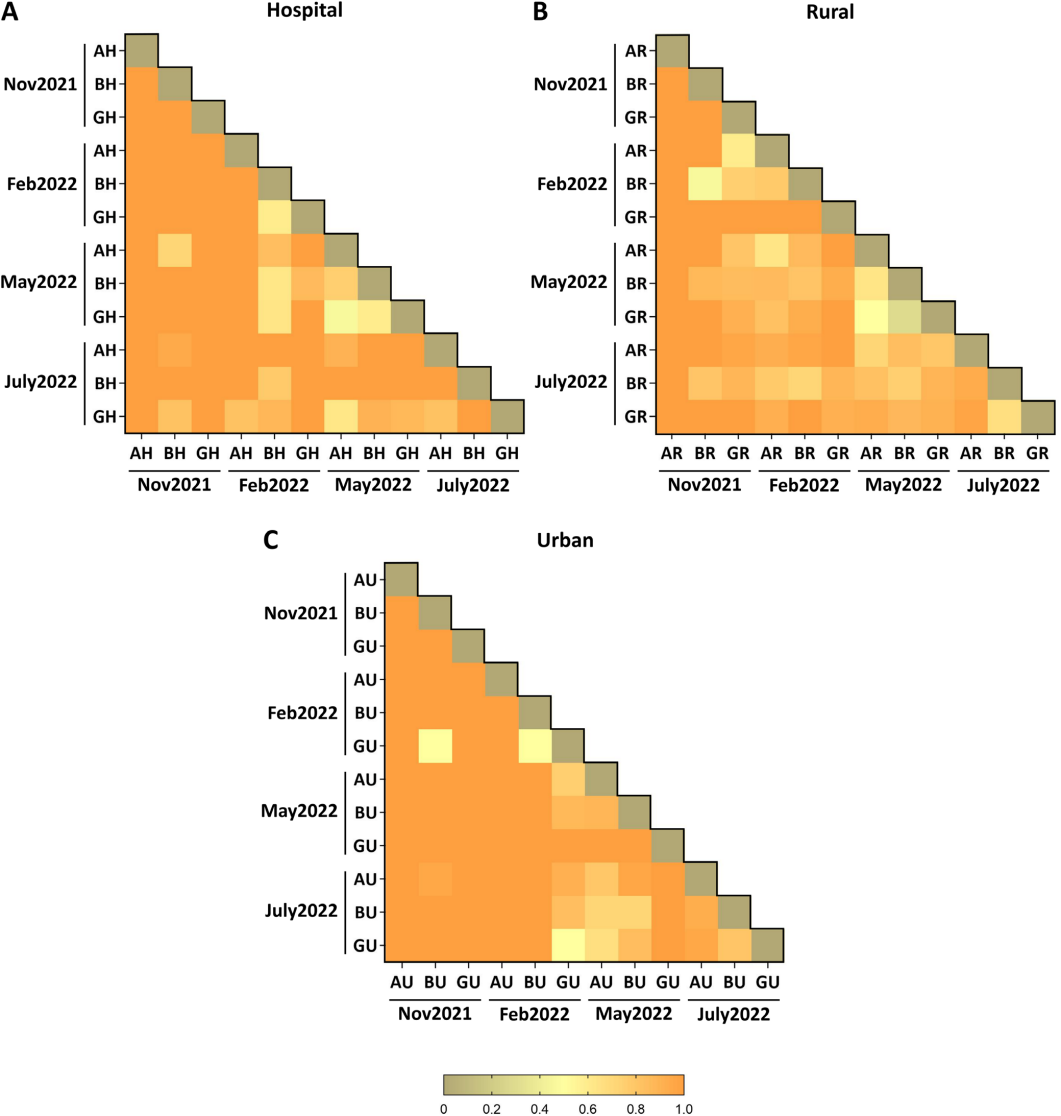
Beta diversity of the identified isolates regarding species abundance by area in each sampling point. Bray–Curtis dissimilarity is represented for hospital (A), rural (B) and urban (C) areas. AH, Araba Hospital; AR, Araba Rural; AU, Araba Urban; BH, Bizkaia Hospital; BR, Bizkaia Rural; BU, Bizkaia Urban; GH, Gipuzkoa Hospital; GR, Gipuzkoa Rural; GU, Gipuzkoa Urban.

The analysis of species diversity in every sampling point individually shows the evolution of potentially antifungal‐resistant mycobiota in the three sampling areas during seasonal changes (Figure 6). The low number of isolates in some samples explained the absence of some Shannon index values (Table 1). Concerning the hospital surrounding area, it can be observed that Araba sampling points had the lower alpha diversity (Table 1). Except in samples from Gipuzkoa in July2022, Araba and Bizkaia showed also decreasing Shannon index trends (Table 1), which could be related to the higher number of isolates of the most abundant genera species (Figure 3B). Most Bray–Curtis Dissimilarity values were above 0.80 (orange), which means that samples were not similar to each other regarding species abundance (Figure 6A). The rural area presented the higher alpha diversity variation as it registered both the higher and lower Shannon index values for each province (Table 1). Regarding beta diversity, the majority of the values of rural area samples were above 0.7 (light orange), representing less dissimilarity between provinces than in both the hospital surrounding and urban areas (Figure 6B). At last, urban area sampling points were more homogeneous in alpha and beta diversity (Table 1 and Figure 6C). Moreover, this area showed the higher beta diversity values, especially when Nov2021 and Feb2022 were compared (Figure 6C). The analysis of PCoA showed that Bizkaia and Gipuzkoa samples exhibited similar distribution patterns (turquoise green circle), whereas Araba sampling points were less closely related (purple circles) (Figure S5). Besides, most of Nov2021 and Feb2022 sampling points cluster in a specific region (light beige circle), as do samples from May2022 and July2022 (orange circle).

**TABLE 1:** Shannon index of each sampling point in every sampling regarding species abundance.

	Nov2021	Feb2022	May2022	July2022
AH	0	0	0.64	0.13
AR	0	1.10	0.56	0.17
AU	0	0	0	0.42
BH	0	1.79	1.69	0.64
BR	0.95	0.87	1.91	2.52
BU	0	0	1.64	1.73
GH	1.56	1.39	0.95	1.83
GR	0.69	0.69	1.37	2.24
GU	0	1.10	1.74	0

Abbreviations: AH, Araba Hospital; AR, Araba Rural; AU, Araba Urban; BH, Bizkaia Hospital; BR, Bizkaia Rural; BU, Bizkaia Urban; GH, Gipuzkoa Hospital; GR, Gipuzkoa Rural; GU, Gipuzkoa Urban.

## 4 Discussion

This is one of the few works based on the isolation and identification of culturable outdoor airborne fungi with potential antifungal resistance in Spain. The microbial concentration showed variations between 40 and 3670 CFU/m^3^, depending on the sampling point and season, when samples were incubated at environmental T. In other locations in Spain such as Murcia, noticeably lower values have been reported (from 263 up to 567 CFU/m^3^) (Soto et al. 2009), whereas other authors observed similar values in Badajoz; ranges went from 5 to 2590 CFU/m^3^ (Fernández‐Rodríguez et al. 2014). Numerous air samplings have also been performed worldwide with variable results, but with similar detection ranges. For instance, values from 113.5 to 1526 CFU/m^3^ were detected in India (Adhikari et al. 2004). However, these ranges may vary depending on the sampling system used, as seen in recent studies conducted in China, where values from 0 to 3224.13 CFU/m^3^ were recorded using the open plate method during winter (Nageen et al. 2021) and maximum values at 340 CFU/m^3^ using the impaction method during 1‐year sampling (Nageen et al. 2023).

Additionally, the present research showed seasonal variations, which have been reported by other authors (Anees‐Hill et al. 2022; Grinn‐Gofroń et al. 2011; Núñez et al. 2021; Picornell et al. 2022). The highest colony concentration was collected in summer, followed by spring, whereas the lowest was detected in autumn. However, other trends were observed in a 7‐year period analysis of anamorphic fungal spores in Madeira (Portugal) (Sousa et al. 2016) and in a 1‐year study in Madrid (Herrero et al. 2006), where the density was higher in spring and autumn. No significant correlation was detected with environmental factors, although a general positive and negative trend was observed with respect to T and RH, respectively. It has also been demonstrated that other factors such as geographical location, the presence of vegetation and anthropological activities have an impact on the fluctuation of these microbial concentrations (Adhikari et al. 2004; Grinn‐Gofroń et al. 2020; Pearson et al. 2015). This work shows that rural locations, which have more vegetation than urban or hospital areas, reported a higher mean CFU/m^3^ value, being more noticeable in July (Figure 2). Additionally, agriculture could also have an impact on this value. In Taiwan, when both rural and urban areas were analysed, authors concluded also that, overall, fungal concentration was higher in rural areas (Lin et al. 2018). Similar results were obtained in Poland for spores measurement (Kasprzyk and Worek 2006) as well as in Portugal (Oliveira et al. 2010).

Few environmental fungi are capable of growing at Ts similar to those of the human body, and in our study, we observed a minimum 30‐fold decrease in the number of colonies detected when samples were incubated at 37°C. Furthermore, the increasing antifungal resistance all over the world has highlighted the need to assess the prevalence of potentially resistant fungal species. For that, we additionally used SAB plates supplemented with VCZ, the first‐line therapy for numerous fungal infections. Despite the selective pressure exerted by the antifungal, the number of CFU/m^3^ at 37°C was reduced to one‐third of its value without VCZ. This suggests that fungi capable of growing at 37°C may exhibit a concerning potential resistance to triazoles. In this last condition, ITS sequencing enabled us to identify all potentially resistant fungal isolates, which belonged to 21 genera and 55 species (Table S3).

Regarding fungal identification, numerous studies have shown that *Cladosporium*, *Alternaria*, *Penicillium* and *Aspergillus* genera are the most common fungi in outdoor environments (Adhikari et al. 2004; Fernández‐Rodríguez et al. 2014; Nageen et al. 2023; Shelton et al. 2002; Soto et al. 2009). Interestingly, no *Cladosporium* species were identified in this study. This could be explained by a combination of higher incubation T (37°C) as well as the presence of the antifungal. The optimal growth T of this genus is around 25°C and most species are not able to grow above 35°C (Bensch et al. 2012; Zalar et al. 2007). In our study, *Alternaria* was the dominant genus (62.31%) (Figure 3A) in SAB‐VCZ plates incubated at 37°C. In agreement with this result, other environmental studies reported this genus as prevalent in Spain (Elvira‐Rendueles et al. 2013; Fernández‐Rodríguez et al. 2014; Soto et al. 2009) and around the globe (Nageen et al. 2023; Sousa et al. 2016). *A. infectoria* was the only species isolated from every sampling location and *A. alternata* was the second most abundant species (Figure 4). The former is the most common clinical species of the genus and it is the causal agent of alternariosis (Pastor and Guarro 2008). It has been detected in phaeohyphomycosis cases (Halaby et al. 2001; Lo Porto et al. 2023), both cutaneous (Lo Cascio et al. 2004; Vennewald and Wollina 2005) and ocular (Ferrer and Alió 2011) infections caused by pigmented fungi. The second species is a prevalent cause of sensitisation in allergic patients (Hernandez‐Ramirez et al. 2021; López Couso et al. 2021; Sánchez et al. 2022) and is related to airway diseases, including chronic rhinosinusitis (Didehdar et al. 2021), asthma (Bush and Prochnau 2004) as well as allergic bronchopulmonary mycosis (Chowdhary, Agarwal, et al. 2012). In addition, it is also known to cause the same infections as *A. infectoria* (Ferrer and Alió 2011; Lo Porto et al. 2023; Vennewald and Wollina 2005). The isolates from this study were able to grow in the presence of the drug VCZ, so infections caused by these strains could represent a serious risk to debilitated patients. Besides, some *Alternaria* species, including the abovementioned ones, are also known plant pathogens (De Mers 2022; Tralamazza et al. 2018). The infection of crops such as cereals causes significant socioeconomic losses globally, and this problem could worsen due to increasing antifungal resistance.

On the other hand, *Talaromyces* was the second most abundant genus and those species represented around 20% of the identified potentially resistant isolates (Figure 3A). Recently, it was reported as the fourth most prevalent genus in a 1‐year sampling research in Tianjin, China (Nageen et al. 2023), but, overall, its prevalence in the environment seems to be low and it has been mostly detected in dust presence (Pyrri et al. 2023; Tajiki et al. 2022; Yarahmadi et al. 2020). Nevertheless, it is important to highlight that it has been confirmed the misclassification of some *Talaromyces* species due to the similarity between this genus and *Penicillium* (Yilmaz et al. 2014). Microscopic identification as well as low‐curated databases could have contributed to this issue, but the use of different approaches, including molecular tools, has helped to solve the problem. In fact, in the latest years, numerous species have been reclassified and added to the genus list (Samson et al. 2011; Visagie et al. 2024; Yilmaz et al. 2014). *Talaromyces* genus is important in the food industry (EFSA Panel on Food Contact Materials, Enzymes and Processing Aids (CEP) et al. 2023; Soliman et al. 2022) as well as in biotechnological processes (Moriwaki‐Takano et al. 2021; Zhang et al. 2017). However, some species of the genus are known to be pathogenic such as *Talaromyces marneffei* , which causes talaromycosis, an invasive infection endemic in Asia (Wang et al. 2023). In the present study, *T. cecidicola* was the most common species from the genus and was exclusive from Bizkaia (Figure S3). Originally, it was isolated from insect galls on scrub oaks in the United States and firstly described as *Penicillium cecidicola* (Seifert et al. 2004). However, this species has not been deeply studied and based on the literature it does not seem to have clinical relevance, although in our study it appears to exhibit potential resistance to azoles.

The abundance of these dominant species affected the diversity detected in the samplings as shown in Table 1. Overall, Bizkaia and Gipuzkoa samples were more similar to each other, with higher alpha diversity than Araba samples, which showed an absence of isolates in samples, reflected in their lower alpha diversity. A possible explanation could be their geographic distribution, which is directly related to their climate.

Regarding areas, a higher Shannon index was observed in rural samples (Table 1), although the overall value was similar for both rural and urban areas (Figure 3D). In fact, *Alternaria* spp. showed similar prevalence in both areas, as well as it happened with *Talaromyces* spp. (Figure 3D). Other authors have reported that *Alternaria* spp. is one of the most abundant genera in urban locations (Kasprzyk and Worek 2006; Nageen et al. 2023; Soto et al. 2009). Additionally, we also analysed hospital surrounding areas, which in all provinces were urban locations. Overall, all areas showed similar beta diversity in species with potential antifungal resistance; although it should be noted that in colder months, samples were different regarding species abundance (Figure 6). Similarly, this could be explained by the increasing *Alternaria* abundance in spring and summer.

Finally, potentially antifungal‐resistant species that belonged to important opportunistic pathogens were detected in all areas: mostly *Alternaria*, *Aspergillus*, *Rhizomucor* and *Rhizopus* species. However, in hospital surrounding area also less common species were identified such as *Lichtheimia ramosa* and *Syncephalastrum racemosum* . This location showed higher alpha diversity and fungal genera richness as shown in Figure 3D. These 14 genera included species of the dominant genera, *Alternaria* (60.47%) and *Talaromyces* (10.47%), but also of *Coprinopsis* (5.81%), *Rhizomucor* and *Rhizopus* (4.65%), *Aspergillus*, *Paecilomyces* and *Penicillium* (2.33%) genera. Other authors have also detected *Aspergillus*, *Coprinopsis* and *Paecilomyces* species, among others, in plates supplemented with itraconazole, when air samples from Seoul were collected (Lee et al. 2015). Regarding health facilities, *Alternaria*, *Aspergillus*, *Cladosporium* and *Penicillium* were reported as common genera in Spain (López et al. 2024), whereas in Portugal *Penicillium* and *Aspergillus* were the most abundant (Cabo Verde et al. 2015). Moreover, meta‐genomic sequencing results from a study in Beijing showed high fungal diversity in hospital environments and authors highlighted the high prevalence of *Aspergillus* species, specifically, *A. fumigatus* (Tong et al. 2017). In fact, numerous studies have focused on detecting this fungal genus in hospitals (Martínez‐Herrera et al. 2016) as well as in outdoor and indoor environments (Guinea et al. 2006; Nafis et al. 2023). This species is known to cause infections that affect mostly immunocompromised patients (Latgé and Chamilos 2019). Besides, this fungus has been recently classified by the World Health Organization as a critical priority fungal pathogen, due to its high mortality rates and increasing resistance to antifungals (World Health Organization 2022). In this study, two potentially resistant *A. fumigatus* isolates were identified in both Araba and Gipuzkoa hospital surrounding area.

To our knowledge, this is the first study to characterise the potentially resistant fungal isolates from the environment of the Basque Country. Despite the limitations in sample size and the absence of antifungal susceptibility results using standardised methods, this work provides valuable information about the fungal genera variability that could be carriers of azole resistance mechanisms in the north of Spain and how species are located in the three distinguished regions of the community. The CFU/m^3^ count on plates incubated with VCZ at 37°C was only 3 times lower than that of plates incubated without VCZ, indicating that fungi capable of growing at 37°C could possess an alarming level of resistance to triazoles, including those that could be potential human pathogens. The highest number of potentially VCZ‐resistant isolates was detected in the warmer months. The provinces of Bizkaia and Gipuzkoa showed similar trends regarding fungal concentration and genera abundance, whereas Araba was more homogeneous overall. Rural areas showed higher potentially resistant fungal concentration in every location and sampling, dominated by *Alternaria*. Moreover, the detection of *Talaromyces* in the present work shows the prevalence of this genus in the environment, with high species richness despite not being usually reported. Besides, it is important to highlight the isolation of fungal pathogens such as *Alternaria* species and other opportunistic fungi as the well‐known *A. fumigatus* . Hospital surrounding area species showed potential risk to immunocompromised patients admitted to health facilities and should be carefully monitored. As we demonstrate in this work, the detection of potentially resistant species from the environment is crucial to know their prevalence and identify possible health risks that may have this origin. Therefore, more surveillance studies are needed to understand and control the increasing fungal resistance rate and to determine its possible relationship to the use of triazoles in agriculture.

## Author Contributions

**Saioa Cendon‐Sanchez:** conceptualization, formal analysis, investigation, methodology, software, writing – original draft, writing – review and editing. **Eduardo Pelegri‐Martinez:** investigation, methodology, writing – review and editing. **Uxue Perez‐Cuesta:** investigation, methodology, writing – review and editing. **Xabier Guruceaga:** investigation, methodology, writing – review and editing. **Andoni Ramirez‐Garcia:** funding acquisition, supervision, writing – review and editing. **Ana Abad‐Diaz‐de‐Cerio:** conceptualization, supervision, writing – review and editing. **Aitor Rementeria:** funding acquisition, conceptualization, writing – review and editing, supervision.

## Ethics Statement

The Ethical Committee from the University of the Basque Country (UPV/EHU) (ref. M20/2020/286) approved all procedures.

## Conflicts of Interest

The authors declare no conflicts of interest.

## Supporting information


**Data S1.** emi470152‐sup‐0001‐supinfo.

**Table S3.** All identified fungal species.

## Data Availability

ITS sequences are available in GenBank under PV369544–PV369864 accession numbers.

## References

[emi470152-bib-0001] Adhikari, A. , M. M. Sen , S. Gupta‐Bhattacharya , and S. Chanda . 2004. “Airborne Viable, Non‐Viable, and Allergenic Fungi in a Rural Agricultural Area of India: A 2‐Year Study at Five Outdoor Sampling Stations.” Science of the Total Environment 326, no. 1–3: 123–141. 10.1016/j.scitotenv.2003.12.007. 15142771

[emi470152-bib-0002] Agarwal, R. , I. S. Sehgal , V. Muthu , et al. 2024. “Revised ISHAM‐ABPA Working Group Clinical Practice Guidelines for Diagnosing, Classifying and Treating Allergic Bronchopulmonary Aspergillosis/Mycoses.” European Respiratory Journal 63, no. 4: 2400061. 10.1183/13993003.00061-2024. 38423624 PMC10991853

[emi470152-bib-0003] Álvarez‐Pérez, S. , M. E. García , E. Martínez‐Nevado , and J. L. Blanco . 2023. “Presence of *Aspergillus fumigatus* With the TR_34_/L98H Cyp51A Mutation and Other Azole‐Resistant Aspergilli in the Air of a Zoological Park.” Research in Veterinary Science 164: 104993. 10.1016/j.rvsc.2023.104993. 37657393

[emi470152-bib-0004] Anees‐Hill, S. , P. Douglas , C. H. Pashley , A. Hansell , and E. L. Marczylo . 2022. “A Systematic Review of Outdoor Airborne Fungal Spore Seasonality Across Europe and the Implications for Health.” Science of the Total Environment 818: 151716. 10.1016/j.scitotenv.2021.151716. 34800445 PMC8919338

[emi470152-bib-0005] Arendrup, M. C. , R. K. Hare , K. M. Jørgensen , et al. 2024. “Environmental Hot Spots and Resistance‐Associated Application Practices for Azole‐Resistant *Aspergillus fumigatus* , Denmark, 2020–2023.” Emerging Infectious Diseases 30, no. 8: 1531–1541. 10.3201/eid3008.240096. 38935978 PMC11286046

[emi470152-bib-0006] Bensch, K. , U. Braun , J. Z. Groenewald , and P. W. Crous . 2012. “The Genus *Cladosporium* .” Studies in Mycology 72, no. 1: 1–401. 10.3114/sim0003. 22815589 PMC3390897

[emi470152-bib-0007] Bush, R. K. , and J. J. Prochnau . 2004. “ *Alternaria* ‐Induced Asthma.” Journal of Allergy and Clinical Immunology 113, no. 2: 227–234. 10.1016/j.jaci.2003.11.023. 14767434

[emi470152-bib-0008] Cabo Verde, S. , S. M. Almeida , J. Matos , et al. 2015. “Microbiological Assessment of Indoor Air Quality at Different Hospital Sites.” Research in Microbiology 166, no. 7: 557–563. 10.1016/j.resmic.2015.03.004. 25869221

[emi470152-bib-0009] Chen, Y. , F. Dong , J. Zhao , et al. 2020. “High Azole Resistance in *Aspergillus fumigatus* Isolates From Strawberry Fields, China, 2018.” Emerging Infectious Diseases 26, no. 1: 81–89. 10.3201/eid2601.190885. 31855142 PMC6924917

[emi470152-bib-0010] Chowdhary, A. , K. Agarwal , H. S. Randhawa , et al. 2012. “A Rare Case of Allergic Bronchopulmonary Mycosis Caused by *Alternaria alternata* .” Medical Mycology 50, no. 8: 890–896. 10.3109/13693786.2012.682320. 22563857

[emi470152-bib-0011] Chowdhary, A. , S. Kathuria , J. Xu , et al. 2012. “Clonal Expansion and Emergence of Environmental Multiple‐Triazole‐Resistant *Aspergillus fumigatus* Strains Carrying the TR_34_/L98H Mutations in the *cyp51A* Gene in India.” PLoS One 7, no. 12: e52871. 10.1371/journal.pone.0052871. 23285210 PMC3532406

[emi470152-bib-0012] Cvetnic, Z. , and S. Pepeljnjak . 1997. “Distribution and Mycotoxin‐Producing Ability of Some Fungal Isolates From the Air.” Atmospheric Environment 31, no. 3: 491–495. 10.1016/S1352-2310(96)00158-6.

[emi470152-bib-0013] De Mers, M. 2022. “ *Alternaria alternata* as Endophyte and Pathogen.” Microbiology 168, no. 3: 001153. 10.1099/mic.0.001153. 35348451 PMC9558358

[emi470152-bib-0014] Didehdar, M. , A. Khoshbayan , S. Vesal , et al. 2021. “An Overview of Possible Pathogenesis Mechanisms of *Alternaria alternata* in Chronic Rhinosinusitis and Nasal Polyposis.” Microbial Pathogenesis 155: 104905. 10.1016/j.micpath.2021.104905. 33930423

[emi470152-bib-0015] EFSA Panel on Food Contact Materials, Enzymes and Processing Aids (CEP) , C. Lambré , J. M. Barat Baviera , et al. 2023. “Safety Evaluation of the Food Enzyme Containing Endo‐Polygalacturonase and Cellulase From the Non‐Genetically Modified *Talaromyces cellulolyticus* Strain NITE BP‐03478.” EFSA Journal 21, no. 2: e07840. 10.2903/j.efsa.2023.7840. 36846396 PMC9951331

[emi470152-bib-0016] Elvira‐Rendueles, B. , J. Moreno , A. Garcia‐Sanchez , N. Vergara , M. J. Martinez‐Garcia , and S. Moreno‐Grau . 2013. “Air‐Spore in Cartagena, Spain: Viable and Non‐Viable Sampling Methods.” Annals of Agricultural and Environmental Medicine 20, no. 4: 664–671. 24364431

[emi470152-bib-0021] Euskalmet . n.d. “The Climate in the Basque Country.” Accessed August 27, 2024. https://www.euskalmet.euskadi.eus/clima/euskadi/.

[emi470152-bib-0017] Euskalmet . 2021. “El Clima en Euskadi.” https://www.euskalmet.euskadi.eus/clima/boletines‐climatologicos/.

[emi470152-bib-0018] Euskalmet . 2022a. “Climatological Report—February 2022.” https://www.euskalmet.euskadi.eus/clima/boletines‐climatologicos/.

[emi470152-bib-0019] Euskalmet . 2022b. “Climatological Report—May 2022.” https://www.euskalmet.euskadi.eus/clima/boletines‐climatologicos/.

[emi470152-bib-0020] Euskalmet . 2022c. “Climatological Report—July 2022.” https://www.euskalmet.euskadi.eus/clima/boletines‐climatologicos/.

[emi470152-bib-0022] EUSTAT . 2024. “Estimated Population of the Basque Country on January 1, According to Historical Territory and Sex. 1976–2024.” Accessed August 29, 2024. https://www.eustat.eus/elementos/ele0011400/poblacion‐estimada‐de‐la‐c‐a‐de‐euskadi‐a‐1‐de‐enero‐segun‐territorio‐historico‐y‐sexo/tbl0011431_c.html.

[emi470152-bib-0023] Fernández‐Rodríguez, S. , R. Tormo‐Molina , J. M. Maya‐Manzano , I. Silva‐Palacios , and Á. Gonzalo‐Garijo . 2014. “Outdoor Airborne Fungi Captured by Viable and Non‐Viable Methods.” Fungal Ecology 7: 16–26. 10.1016/j.funeco.2013.11.004.

[emi470152-bib-0024] Ferrer, C. , and J. L. Alió . 2011. “Evaluation of Molecular Diagnosis in Fungal Keratitis. Ten Years of Experience.” Journal of Ophthalmic Inflammation and Infection 1, no. 1: 15–22. 10.1007/s12348-011-0019-9. 21475656 PMC3062769

[emi470152-bib-0025] Fisher, M. C. , N. J. Hawkins , D. Sanglard , and S. J. Gurr . 2018. “Worldwide Emergence of Resistance to Antifungal Drugs Challenges Human Health and Food Security.” Science 360, no. 6390: 739–742. 10.1126/science.aap7999. 29773744

[emi470152-bib-0026] Fukutomi, Y. , and M. Taniguchi . 2015. “Sensitization to Fungal Allergens: Resolved and Unresolved Issues.” Allergology International 64, no. 4: 321–331. 10.1016/j.alit.2015.05.007. 26433528

[emi470152-bib-0027] Grinn‐Gofroń, A. , T. Çeter , N. M. Pinar , et al. 2020. “Airborne Fungal Spore Load and Season Timing in the Central and Eastern Black Sea Region of Turkey Explained by Climate Conditions and Land Use.” Agricultural and Forest Meteorology 295: 108191. 10.1016/j.agrformet.2020.108191.

[emi470152-bib-0028] Grinn‐Gofroń, A. , A. Strzelczak , and T. Wolski . 2011. “The Relationships Between Air Pollutants, Meteorological Parameters and Concentration of Airborne Fungal Spores.” Environmental Pollution 159, no. 2: 602–608. 10.1016/j.envpol.2010.10.002. 21030122

[emi470152-bib-0029] Guinea, J. , T. Peláez , L. Alcalá , and E. Bouza . 2006. “Outdoor Environmental Levels of *Aspergillus* spp. Conidia Over a Wide Geographical Area.” Medical Mycology 44, no. 4: 349–356. 10.1080/13693780500488939. 16772229

[emi470152-bib-0030] Halaby, T. , H. Boots , A. Vermeulen , et al. 2001. “Phaeohyphomycosis Caused by *Alternaria infectoria* in a Renal Transplant Recipient.” Journal of Clinical Microbiology 39, no. 5: 1952–1955. 10.1128/JCM.39.5.1952-1955.2001. 11326020 PMC88055

[emi470152-bib-0031] Hernandez‐Ramirez, G. , D. Barber , J. Tome‐amat , M. Garrido‐Arandia , and A. Diaz‐Perales . 2021. “ *Alternaria* as an Inducer of Allergic Sensitization.” Journal of Fungi 7, no. 10: 838. 10.3390/jof7100838. 34682259 PMC8539034

[emi470152-bib-0032] Herrero, A. D. , S. S. Ruiz , M. G. Bustillo , and P. C. Morales . 2006. “Study of Airborne Fungal Spores in Madrid, Spain.” Aerobiologia 22: 135–142. 10.1007/s10453-006-9025-z.

[emi470152-bib-0033] Hervás‐Aguilar, A. , J. M. Rodríguez , J. Tilburn , H. N. Arst , and M. A. Peñalva . 2007. “Evidence for the Direct Involvement of the Proteasome in the Proteolytic Processing of the *Aspergillus nidulans* Zinc Finger Transcription Factor PacC.” Journal of Biological Chemistry 282, no. 48: 34735–34747. 10.1074/jbc.M706723200. 17911112

[emi470152-bib-0034] Howard, S. J. , D. Cerar , M. J. Anderson , et al. 2009. “Frequency and Evolution of Azole Resistance in *Aspergillus fumigatus* Associated With Treatment Failure.” Emerging Infectious Diseases 15, no. 7: 1068–1076. 10.3201/eid1507.090043. 19624922 PMC2744247

[emi470152-bib-0035] Kasprzyk, I. , and M. Worek . 2006. “Airborne Fungal Spores in Urban and Rural Environments in Poland.” Aerobiologia 22: 169–176. 10.1007/s10453-006-9029-8.

[emi470152-bib-0036] Latgé, J. P. , and G. Chamilos . 2019. “ *Aspergillus fumigatus* and Aspergillosis in 2019.” Clinical Microbiology Reviews 33, no. 1: e00140‐18. 10.1128/CMR.00140-18. 31722890 PMC6860006

[emi470152-bib-0037] Lee, S. , S. Xu , C. P. Bivila , et al. 2015. “Triazole Susceptibilities in Thermotolerant Fungal Isolates From Outdoor Air in the Seoul Capital Area in South Korea.” PLoS One 10, no. 9: e0138725. 10.1371/journal.pone.0138725. 26405807 PMC4583468

[emi470152-bib-0038] Lin, W. R. , P. H. Wang , C. J. Tien , W. Y. Chen , Y. A. Yu , and L. Y. Hsu . 2018. “Changes in Airborne Fungal Flora Along an Urban to Rural Gradient.” Journal of Aerosol Science 116: 116–123. 10.1016/j.jaerosci.2017.11.010.

[emi470152-bib-0039] Lo Cascio, G. , M. Ligozzi , L. Maccacaro , and R. Fontana . 2004. “Utility of Molecular Identification in Opportunistic Mycotic Infections: A Case of Cutaneous *Alternaria infectoria* Infection in a Cardiac Transplant Recipient.” Journal of Clinical Microbiology 42, no. 11: 5334–5336. 10.1128/JCM.42.11.5334-5336.2004. 15528736 PMC525136

[emi470152-bib-0040] Lo Porto, D. , A. Cona , F. Todaro , et al. 2023. “Phaeohyphomycosis in Solid Organ Transplant Recipients: A Case Series and Narrative Review of the Literature.” Journal of Fungi 9, no. 3: 283. 10.3390/jof9030283. 36983451 PMC10057669

[emi470152-bib-0041] López, A. , E. Fuentes‐Ferragud , M. J. Mora , et al. 2024. “Air Quality of Health Facilities in Spain.” Chemosphere 362: 142615. 10.1016/j.chemosphere.2024.142615. 38880262

[emi470152-bib-0042] López Couso, V. P. , M. Tortajada‐Girbés , D. Rodriguez Gil , J. Martínez Quesada , and R. Palacios Pelaez . 2021. “Fungi Sensitization in Spain: Importance of the *Alternaria alternata* Species and Its Major Allergen Alt a 1 in the Allergenicity.” Journal of Fungi 7, no. 8: 631. 10.3390/jof7080631. 34436170 PMC8398619

[emi470152-bib-0043] Martinez‐Bracero, M. , E. Markey , J. H. Clancy , E. J. McGillicuddy , G. Sewell , and D. J. O'Connor . 2022. “Airborne Fungal Spore Review, New Advances and Automatisation.” Atmosphere 13, no. 2: 308. 10.3390/atmos13020308.

[emi470152-bib-0044] Martínez‐Herrera, E. O. , M. G. Frías‐De‐León , E. Duarte‐Escalante , et al. 2016. “Fungal Diversity and *Aspergillus* Species in Hospital Environments.” Annals of Agricultural and Environmental Medicine 23, no. 2: 264–269. 10.5604/12321966.1203888. 27294630

[emi470152-bib-0045] Moriwaki‐Takano, M. , C. Asada , and Y. Nakamura . 2021. “Production of Spiculisporic Acid by *Talaromyces trachyspermus* in Fed‐Batch Bioreactor Culture.” Bioresources and Bioprocessing 8, no. 1: 59. 10.1186/s40643-021-00414-1. 38650186 PMC10991155

[emi470152-bib-0046] Mortensen, K. L. , E. Mellado , C. Lass‐Flörl , J. L. Rodriguez‐Tudela , H. K. Johansen , and M. C. Arendrup . 2010. “Environmental Study of Azole‐Resistant *Aspergillus fumigatus* and Other Aspergilli in Austria, Denmark, and Spain.” Antimicrobial Agents and Chemotherapy 54, no. 11: 4545–4549. 10.1128/AAC.00692-10. 20805399 PMC2976122

[emi470152-bib-0047] Nafis, M. M. H. , Z. M. Quach , A. A. Q. A. Al‐Shaarani , M. H. M. Muafa , and L. Pecoraro . 2023. “Pathogenicity of *Aspergillus* Airborne Fungal Species Collected From Indoor and Outdoor Public Areas in Tianjin, China.” Pathogens 12, no. 9: 1154. 10.3390/pathogens12091154. 37764962 PMC10534727

[emi470152-bib-0048] Nageen, Y. , M. D. Asemoloye , S. Põlme , et al. 2021. “Analysis of Culturable Airborne Fungi in Outdoor Environments in Tianjin, China.” BMC Microbiology 21, no. 1: 134. 10.1186/s12866-021-02205-2. 33932997 PMC8088404

[emi470152-bib-0049] Nageen, Y. , X. Wang , and L. Pecoraro . 2023. “Seasonal Variation of Airborne Fungal Diversity and Community Structure in Urban Outdoor Environments in Tianjin, China.” Frontiers in Microbiology 13: 1043224. 10.3389/fmicb.2022.1043224. 36699604 PMC9869124

[emi470152-bib-0050] Núñez, A. , A. M. García , D. A. Moreno , and R. Guantes . 2021. “Seasonal Changes Dominate Long‐Term Variability of the Urban Air Microbiome Across Space and Time.” Environment International 150: 106423. 10.1016/j.envint.2021.106423. 33578068

[emi470152-bib-0051] Oliveira, M. , H. Ribeiro , L. Delgado , J. Fonseca , M. G. Castel‐Branco , and I. Abreu . 2010. “Outdoor Allergenic Fungal Spores: Comparison Between an Urban and a Rural Area in Northern Portugal.” Journal of Investigational Allergology and Clinical Immunology 20, no. 2: 117–128. 20461966

[emi470152-bib-0052] Pastor, F. J. , and J. Guarro . 2008. “ *Alternaria* Infections: Laboratory Diagnosis and Relevant Clinical Features.” Clinical Microbiology and Infection 14, no. 8: 734–746. 10.1111/j.1469-0691.2008.02024.x. 18727797

[emi470152-bib-0053] Patterson, T. F. , G. R. Thompson , D. W. Denning , et al. 2016. “Practice Guidelines for the Diagnosis and Management of Aspergillosis: 2016 Update by the Infectious Diseases Society of America.” Clinical Infectious Diseases 63, no. 4: e1–e60. 10.1093/cid/ciw326. 27365388 PMC4967602

[emi470152-bib-0054] Pearson, C. , E. Littlewood , P. Douglas , S. Robertson , T. W. Gant , and A. L. Hansell . 2015. “Exposures and Health Outcomes in Relation to Bioaerosol Emissions From Composting Facilities: A Systematic Review of Occupational and Community Studies.” Journal of Toxicology and Environmental Health—Part B: Critical Reviews 18, no. 1: 43–69. 10.1080/10937404.2015.1009961. 25825807 PMC4409048

[emi470152-bib-0055] Picornell, A. , J. Rojo , M. M. Trigo , et al. 2022. “Environmental Drivers of the Seasonal Exposure to Airborne *Alternaria* Spores in Spain.” Science of the Total Environment 823: 153596. 10.1016/j.scitotenv.2022.153596. 35122844

[emi470152-bib-0056] Pyrri, I. , A. Stamatelopoulou , D. Pardali , and T. Maggos . 2023. “The Air and Dust Invisible Mycobiome of Urban Domestic Environments.” Science of the Total Environment 904: 16628. 10.1016/j.scitotenv.2023.1662282023. 37591388

[emi470152-bib-0057] Sabariego, S. , A. Díez , and M. Gutiérrez . 2007. “Monitoring of Airborne Fungi in Madrid (Spain).” Acta Botanica Croatica 66, no. 2: 117–126.

[emi470152-bib-0058] Samson, R. A. , N. Yilmaz , J. Houbraken , et al. 2011. “Phylogeny and Nomenclature of the Genus *Talaromyces* and Taxa Accommodated in *Penicillium* Subgenus *Biverticillium* .” Studies in Mycology 70, no. 1: 159–183. 10.3114/sim.2011.70.04. 22308048 PMC3233910

[emi470152-bib-0059] Sánchez, P. , A. Vélez‐Del‐burgo , E. Suñén , J. Martínez , and I. Postigo . 2022. “Fungal Allergen and Mold Allergy Diagnosis: Role and Relevance of *Alternaria alternata* Alt a 1 Protein Family.” Journal of Fungi 8, no. 3: 277. 10.3390/jof8030277. 35330279 PMC8954643

[emi470152-bib-0060] Sandoval‐Denis, M. , D. A. Sutton , A. Martin‐Vicente , et al. 2015. “ *Cladosporium* Species Recovered From Clinical Samples in the United States.” Journal of Clinical Microbiology 53, no. 9: 2990–3000. 10.1128/JCM.01482-15. 26179305 PMC4540897

[emi470152-bib-0061] Seifert, K. A. , E. S. Hoekstra , J. C. Frisvad , and G. Louis‐Seize . 2004. “ *Penicillium cecidicola* , a New Species on Cynipid Insect Galls on *Quercus pacifica* in the Western United States.” Studies in Mycology 50: 517–523.

[emi470152-bib-0062] Shelton, B. G. , K. H. Kirkland , W. D. Flanders , and G. K. Morris . 2002. “Profiles of Airborne Fungi in Buildings and Outdoor Environments in the United States.” Applied and Environmental Microbiology 68, no. 4: 1743–1753. 10.1128/AEM.68.4.1743-1753.2002. 11916692 PMC123871

[emi470152-bib-0063] Snelders, E. , S. M. T. Camps , A. Karawajczyk , et al. 2012. “Triazole Fungicides Can Induce Cross‐Resistance to Medical Triazoles in *Aspergillus fumigatus* .” PLoS One 7, no. 3: e31801. 10.1371/journal.pone.0031801. 22396740 PMC3291550

[emi470152-bib-0064] Snelders, E. , R. A. G. Huis In't Veld , A. J. M. M. Rijs , G. H. J. Kema , W. J. G. Melchers , and P. E. Verweij . 2009. “Possible Environmental Origin of Resistance of *Aspergillus fumigatus* to Medical Triazoles.” Applied and Environmental Microbiology 75, no. 12: 4053–4057. 10.1128/AEM.00231-09. 19376899 PMC2698372

[emi470152-bib-0065] Soliman, I. A. , Y. A. Hasanien , A. G. Zaki , H. A. Shawky , and A. A. Nassrallah . 2022. “Irradiation Impact on Biological Activities of Anthraquinone Pigment Produced From *Talaromyces purpureogenus* and Its Evaluation, Characterization and Application in Beef Burger as Natural Preservative.” BMC Microbiology 22, no. 1: 325. 10.1186/s12866-022-02734-4. 36581795 PMC9801527

[emi470152-bib-0066] Soto, T. , M. Lozano , J. Vicente‐Soler , J. Cansado , and M. Gacto . 2009. “Microbiological Survey of the Aerial Contamination in Urban Areas of the City of Murcia, Spain.” Anales de Biologia 31: 7–13.

[emi470152-bib-0067] Sousa, L. , I. C. Camacho , A. Grinn‐Gofroń , and R. Camacho . 2016. “Monitoring of Anamorphic Fungal Spores in Madeira Region (Portugal), 2003–2008.” Aerobiologia 32: 303–315. 10.1007/s10453-015-9400-8.

[emi470152-bib-0068] Tajiki, F. , H. M. Asgari , I. Zamani , and F. Ghanbari . 2022. “Assessing the Relationship Between Airborne Fungi and Potential Dust Sources Using a Combined Approach.” Environmental Science and Pollution Research International 29, no. 12: 17799–17810. 10.1007/s11356-021-17028-x. 34676476

[emi470152-bib-0069] Tangwattanachuleeporn, M. , N. Minarin , S. Saichan , et al. 2017. “Prevalence of Azole‐Resistant *Aspergillus fumigatus* in the Environment of Thailand.” Medical Mycology 55, no. 4: 429–435. 10.1093/mmy/myw090. 27664994

[emi470152-bib-0070] Tashiro, M. , K. Izumikawa , K. Hirano , et al. 2012. “Correlation Between Triazole Treatment History and Susceptibility in Clinically Isolated *Aspergillus fumigatus* .” Antimicrobial Agents and Chemotherapy 56, no. 9: 4870–4875. 10.1128/AAC.00514-12. 22751542 PMC3421857

[emi470152-bib-0071] Toda, M. , K. D. Beer , K. M. Kuivila , T. M. Chiller , and B. R. Jackson . 2021. “Trends in Agricultural Triazole Fungicide Use in the United States, 1992–2016 and Possible Implications for Antifungal‐Resistant Fungi in Human Disease.” Environmental Health Perspectives 129, no. 5: 55001. 10.1289/EHP7484. 33949891 PMC8098123

[emi470152-bib-0072] Tong, X. , H. Xu , L. Zou , et al. 2017. “High Diversity of Airborne Fungi in the Hospital Environment as Revealed by Meta‐Sequencing‐Based Microbiome Analysis.” Scientific Reports 7: 39606. 10.1038/srep39606. 28045065 PMC5206710

[emi470152-bib-0073] Tralamazza, S. M. , K. C. Piacentini , C. H. T. Iwase , and L. d. O. Rocha . 2018. “Toxigenic *Alternaria* Species: Impact in Cereals Worldwide.” Current Opinion in Food Science 23: 57–63. 10.1016/j.cofs.2018.05.002.

[emi470152-bib-0074] Tsitsopoulou, A. , R. Posso , L. Vale , S. Bebb , E. Johnson , and P. L. White . 2018. “Determination of the Prevalence of Triazole Resistance in Environmental *Aspergillus fumigatus* Strains Isolated in South Wales, UK.” Frontiers in Microbiology 9: 1395. 10.3389/fmicb.2018.01395. 29997605 PMC6028733

[emi470152-bib-0075] Vélez‐Pereira, A. M. , C. De Linares , R. Delgado , and J. Belmonte . 2016. “Temporal Trends of the Airborne Fungal Spores in Catalonia (NE Spain), 1995–2013.” Aerobiologia 32: 23–37. 10.1007/s10453-015-9410-6.

[emi470152-bib-0076] Vennewald, I. , and U. Wollina . 2005. “Cutaneous Infections due to Opportunistic Molds: Uncommon Presentations.” Clinics in Dermatology 23, no. 6: 565–571. 10.1016/j.clindermatol.2005.01.003. 16325064

[emi470152-bib-0077] Visagie, C. M. , N. Yilmaz , S. Kocsubé , et al. 2024. “A Review of Recently Introduced *Aspergillus*, *Penicillium*, *Talaromyces* and Other *Eurotiales* Species.” Studies in Mycology 107: 1–66. 10.3114/sim.2024.107.01. 38600958 PMC11003441

[emi470152-bib-0078] Walsh, T. J. , E. J. Anaissie , D. W. Denning , et al. 2008. “Treatment of Aspergillosis: Clinical Practice Guidelines of the Infectious Diseases Society of America.” Clinical Infectious Diseases 46, no. 3: 327–360. 10.1086/525258. 18177225

[emi470152-bib-0079] Wang, F. , R. H. Han , and S. Chen . 2023. “An Overlooked and Underrated Endemic Mycosis—Talaromycosis and the Pathogenic Fungus *Talaromyces marneffei* .” Clinical Microbiology Reviews 36, no. 1: e0005122. 10.1128/cmr.00051-22. 36648228 PMC10035316

[emi470152-bib-0080] White, T. J. , T. Bruns , S. Lee , and J. Taylor . 1990. “Amplification and Direct Sequencing of Fungal Ribosomal RNA Genes for Phylogenetis.” In PCR Protocols: A Guide to Methods and Applications, edited by M. A. Innis , D. H. Gelfand , J. J. Sninsky , and T. J. White , 315–322. Academic Press, Inc.

[emi470152-bib-0081] World Health Organization . 2022. “WHO Fungal Priority Pathogens List to Guide Research, Development and Public Health Action.” *World Health Organization*.

[emi470152-bib-0082] Yarahmadi, M. , S. J. Hashemi , A. Sepahvand , et al. 2020. “Evaluation of Phenotypes and Genotypes of Airborne Fungi During Middle Eastern Dust Storms.” Journal of Environmental Health Science and Engineering 18, no. 1: 11–20. 10.1007/s40201-019-00428-0. 32399217 PMC7203330

[emi470152-bib-0083] Yilmaz, N. , C. M. Visagie , J. Houbraken , J. C. Frisvad , and R. A. Samson . 2014. “Polyphasic Taxonomy of the Genus *Talaromyces* .” Studies in Mycology 78: 175–341. 10.1016/j.simyco.2014.08.001. 25492983 PMC4255554

[emi470152-bib-0084] Zalar, P. , G. S. De Hoog , H. J. Schroers , P. W. Crous , J. Z. Groenewald , and N. Gunde‐Cimerman . 2007. “Phylogeny and Ecology of the Ubiquitous Saprobe *Cladosporium sphaerospermum* , With Descriptions of Seven New Species From Hypersaline Environments.” Studies in Mycology 58: 157–183. 10.3114/sim.2007.58.06. 18490999 PMC2104741

[emi470152-bib-0085] Zhang, Y. Q. , R. H. Li , H. B. Zhang , M. Wu , and X. Q. Hu . 2017. “Purification, Characterization, and Application of a Thermostable Dextranase From *Talaromyces pinophilus* .” Journal of Industrial Microbiology and Biotechnology 44, no. 2: 317–327. 10.1007/s10295-016-1886-8. 28013394

