# Supplementary material for: Identification and Distribution of Potentially Azole‐Resistant Airborne Fungi in Outdoor Environments of the Basque Country
Source: Environ Microbiol Rep. 2025 Oct 29;17(6):e70152. doi: 10.1111/1758-2229.70152 (PMC12571512; doi:10.1111/1758-2229.70152)
Supplement: Supplementary file 1 — Data S1. emi470152‐sup‐0001‐supinfo. [file EMI4-17-e70152-s001.docx]

**TABLES**

**Table S1. Temperature (T) and relative humidity (RH) for every sampling point in each sampling.**

|  |  | **AH^a^** | **AR** | **AU** | **BH** | **BR** | **BU** | **GH** | **GR** | **GU** |
| --- | --- | --- | --- | --- | --- | --- | --- | --- | --- | --- |
| **Nov2021** | **T (°C)** | 11 | 10 | 12 | 7 | 9 | 6 | 13 | 11 | 13 |
|  | **RH (%)** | 77 | 78 | 74 | 88 | 74 | 92 | 73 | 76 | 72 |
| **Feb2022** | **T (°C)** | 15 | 15 | 16 | 6 | 7 | 6 | 13 | 10 | 16 |
|  | **RH (%)** | 57 | 57 | 56 | 80 | 73 | 77 | 75 | 75 | 50 |
| **May2022** | **T (°C)** | 25 | 24 | 25 | 13 | 16 | 12 | 20 | 23 | 21 |
|  | **RH (%)** | 42 | 45 | 42 | 82 | 75 | 79 | 51 | 64 | 50 |
| **July2022** | **T (°C)** | 34 | 31 | 35 | 19 | 23 | 19 | 25 | 24 | 27 |
|  | **RH (%)** | 29 | 28 | 25 | 83 | 70 | 79 | 61 | 60 | 57 |

^a^ AH: Araba Hospital. AR: Araba Rural. AU: Araba Urban. BH: Bizkaia Hospital. BR: Bizkaia Rural. BU: Bizkaia Urban. GH: Gipuzkoa Hospital. GR: Gipuzkoa Rural. GU: Gipuzkoa Urban.

**Table S2. Spearman correlation analysis between CFU/m^3^ and environmental factors, temperature and humidity (RH), in every sampling point.**

|  |  | **AH^a^** | **AR** | **AU** | **BH** | **BR** | **BU** | **GH** | **GR** | **GU** |
| --- | --- | --- | --- | --- | --- | --- | --- | --- | --- | --- |
| **25 °C** | **T** | 0.80 | 1.00** | 0.40 | 0.60 | 0.40 | 0.74 | 0.32 | 0.80 | 0.40 |
|  | **RH** | -0.80 | -1.00** | -0.40 | -0.40 | -0.80 | -0.32 | 0.40 | -0.40 | 0.21 |
| **37 °C** | **T** | 1.00** | 1.00** | 0.40 | 0.00 | 0.80 | 0.95 | -0.74 | 1.00** | -0.80 |
|  | **RH** | -1.00** | -1.00** | -0.40 | -0.80 | -0.40 | -0.32 | 0.80 | -0.80 | 0.63 |
| **VCZ 37 °C** | **T** | 1.00** | 1.00** | 0.80 | 0.00 | 0.60 | 0.78 | 0.63 | 0.95 | -0.63 |
|  | **RH** | -1.00** | -1.00** | -0.80 | -0.80 | 0.00 | 0.32 | -0.40 | -0.95 | -0.50 |

** significant correlation p < 0.01 (bilateral)

^a^ AH: Araba Hospital. AR: Araba Rural. AU: Araba Urban. BH: Bizkaia Hospital. BR: Bizkaia Rural. BU: Bizkaia Urban. GH: Gipuzkoa Hospital. GR: Gipuzkoa Rural. GU: Gipuzkoa Urban.

**FIGURES**


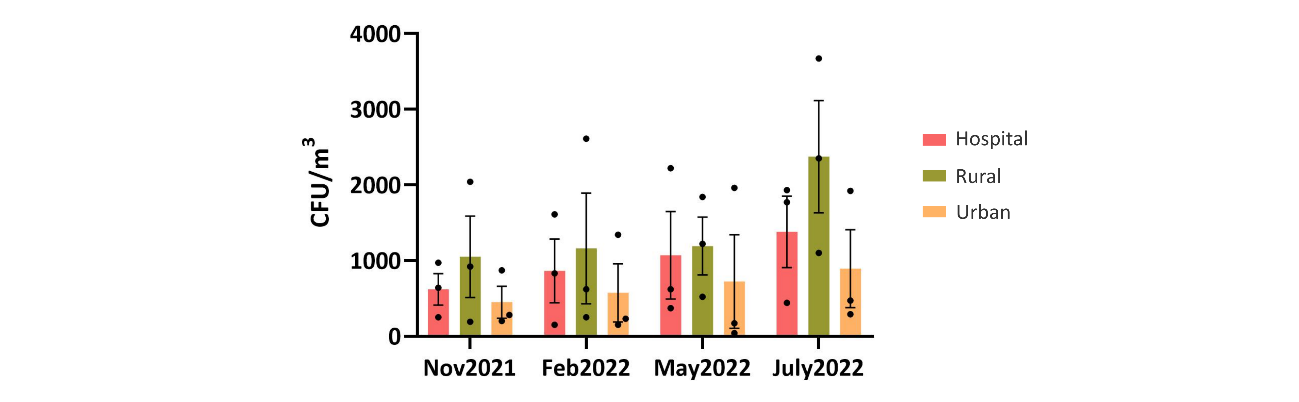


**Figure S1.** **Total counts of colony forming units per volume of filtered air (CFU/m^3^) on Sabouraud plates incubated at 25°C.** Data for each sampling have been grouped by areas. Each area is shown in a color.


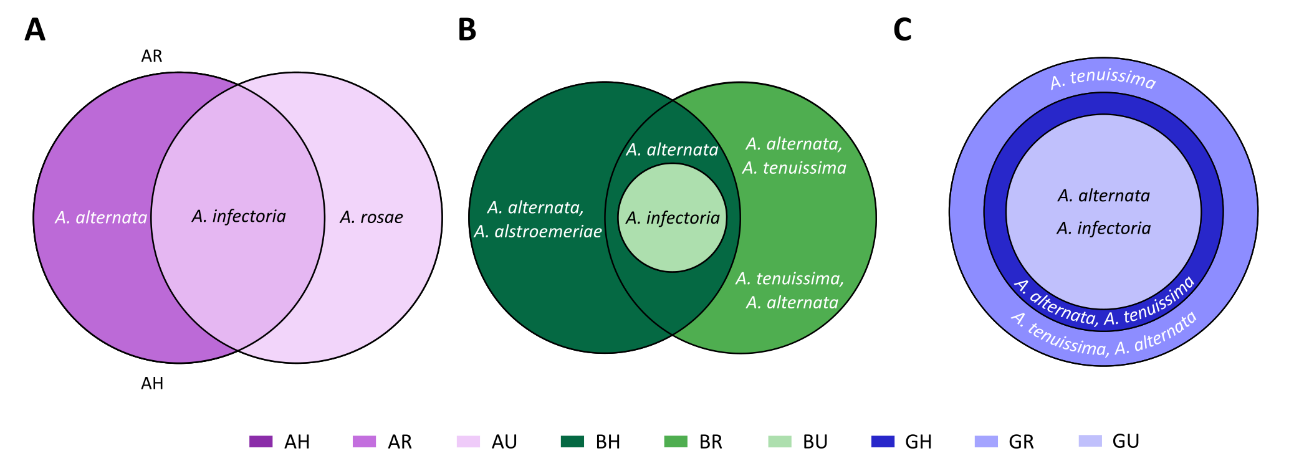


**Figure S2. *Alternaria* spp. Venn diagrams by province.** Distribution of the species in Araba (A), Bizkaia (B) and Gipuzkoa (C). Each sampling point is shown in different shades of color purple (Araba), green (Bizkaia) or blue (Gipuzkoa). AH: Araba Hospital. AR: Araba Rural. AU: Araba Urban. BH: Bizkaia Hospital. BR: Bizkaia Rural. BU: Bizkaia Urban. GH: Gipuzkoa Hospital. GR: Gipuzkoa Rural. GU: Gipuzkoa Urban.

**
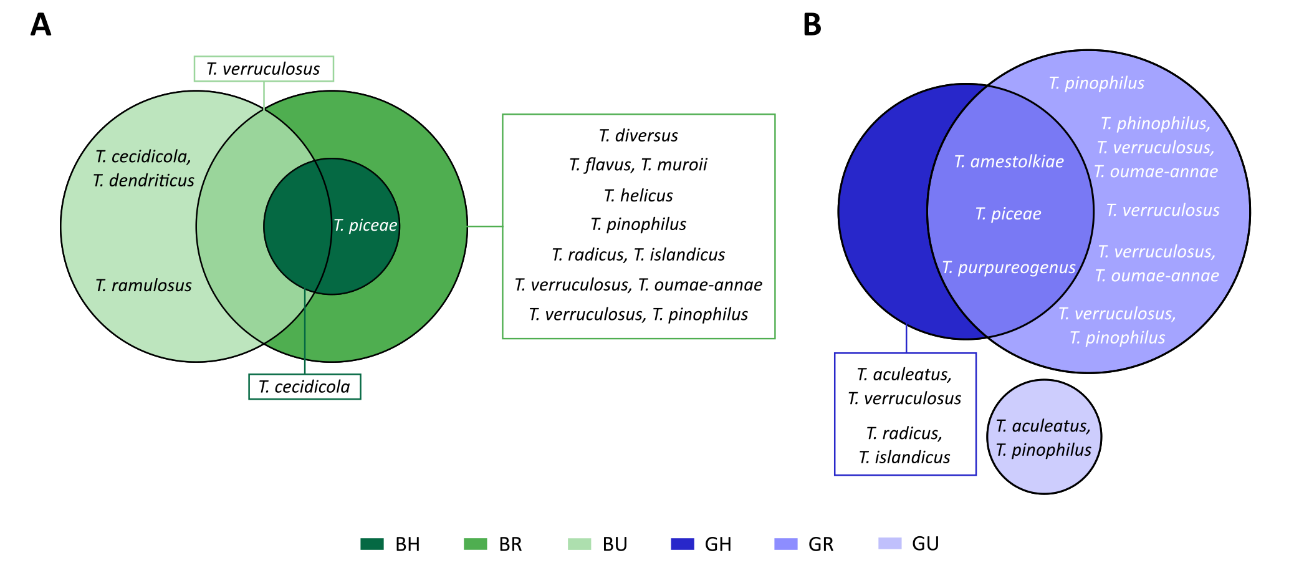
**

**Figure S3. *Talaromyces* spp. Venn diagrams by province.** Distribution of the species in Bizkaia (A) and Gipuzkoa (B). Each sampling point is shown in different shades of color purple (Araba), green (Bizkaia) or blue (Gipuzkoa). BH: Bizkaia Hospital. BR: Bizkaia Rural. BU: Bizkaia Urban. GH: Gipuzkoa Hospital. GR: Gipuzkoa Rural. GU: Gipuzkoa Urban.


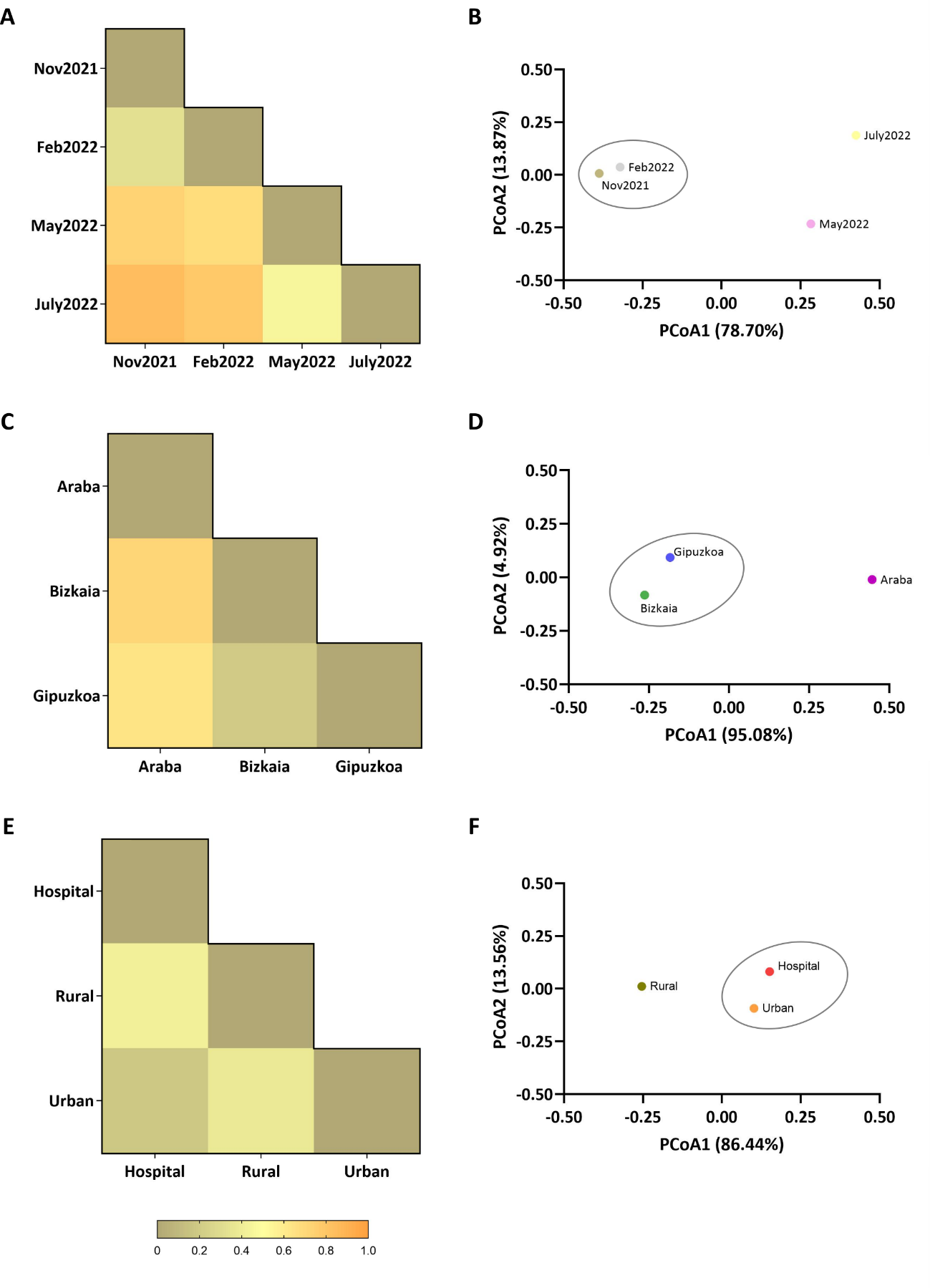


**Figure S4. Diversity of the identified isolates regarding genera abundance.** Bray-Curtis dissimilarity (A, C, E) and PCoA (B, D, F) are shown. Each row represents an aggrupation: sampling, province and area, respectively. Groups are distinguished by different colors.


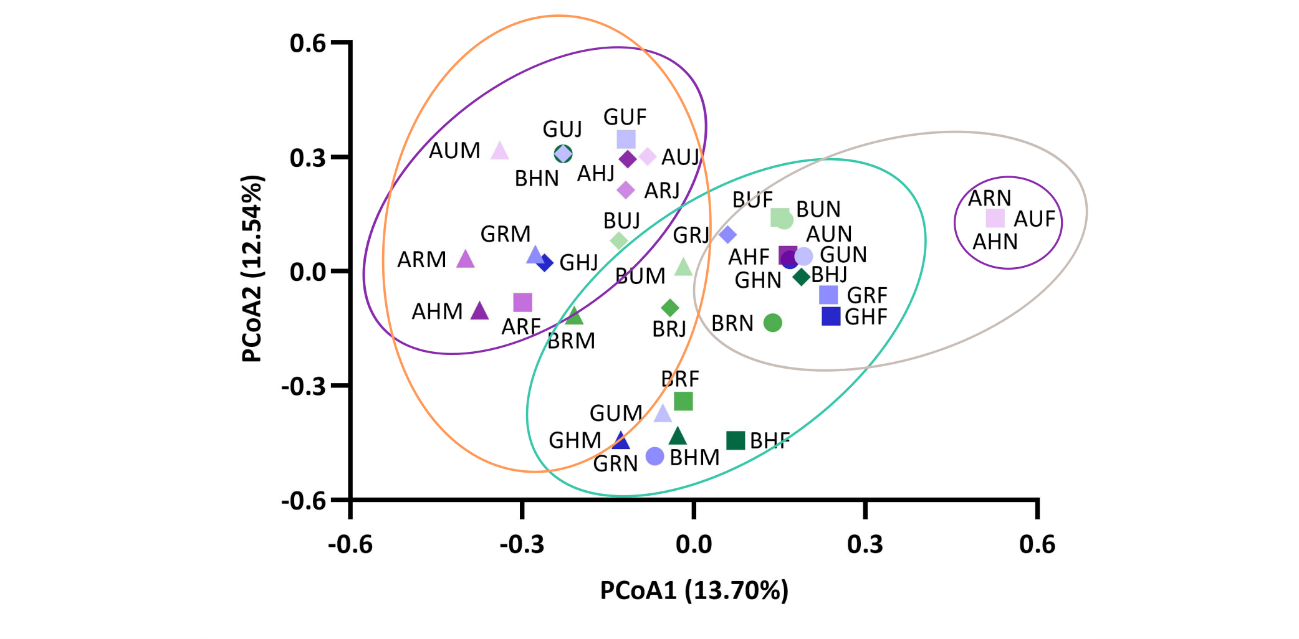


**Figure S5. PCoA analysis regarding species abundance.** Each sampling point is shown in different shades of color: purple (Araba), green (Bizkaia) or blue (Gipuzkoa). AH: Araba Hospital. AR: Araba Rural. AU: Araba Urban. BH: Bizkaia Hospital. BR: Bizkaia Rural. BU: Bizkaia Urban. GH: Gipuzkoa Hospital. GR: Gipuzkoa Rural. GU: Gipuzkoa Urban. The last letter in PCoA points indicates the sampling: N for Nov2021, F for Feb2022, M for May2022 and J for July2022.
