# Supplementary material for: Identification and Distribution of Potentially Azole‐Resistant Airborne Fungi in Outdoor Environments of the Basque Country
Source: Environ Microbiol Rep. 2025 Oct 29;17(6):e70152. doi: 10.1111/1758-2229.70152 (PMC12571512; doi:10.1111/1758-2229.70152)
Supplement: Supplementary file 2 — Table S3. All identified fungal species. [file EMI4-17-e70152-s002.docx]

**Table S3. All identified fungal species.**

| **Species** | **Counts^a^** | **Localization^b^** | **Sampling** | **Percent identity^c^** |
| --- | --- | --- | --- | --- |
| *Talaromyces purpureogenus, Talaromyces atroroseus* | 1 | AU | Nov2021 | 100%, 99.82% |
| *Alternaria infectoria* | 1 | BH | Nov2021 | 99.64% |
| *Talaromyces cecidicola* | 3 | BR | Nov2021 | 99.35% |
| *Talaromyces flavus, Talaromyces muroii* | 1 | BR | Nov2021 | 99.63%, 99.25% |
| *Talaromyces helicus* | 1 | BR | Nov2021 | 98.40% |
| *Penicillium citrinum* | 1 | BU | Nov2021 | 98.45% |
| *Alternaria alternata, Alternaria tenuissima* | 1 | GH | Nov2021 | 100% |
| *Curvularia penniseti* | 1 | GH | Nov2021 | 99.63% |
| *Penicillium capsulatum* | 2 | GH | Nov2021 | 99.82% |
| *Rasamsonia argillacea* | 1 | GH | Nov2021 | 99.22% |
| *Talaromyces radicus, Talaromyces islandicus* | 1 | GH | Nov2021 | 99.64%, 97.14% |
| *Alternaria alternata* | 1 | GR | Nov2021 | 100% |
| *Coprinopsis cinerea* | 1 | GR | Nov2021 | 99.69% |
| *Talaromyces aculeatus, Talaromyces pinophilus* | 2 | GU | Nov2021 | 97.04%, 96.25% |
| *Aspergillus fumigatus* | 1 | AH | Feb2022 | 98.06% |
| *Alternaria alternata* | 1 | AR | Feb2022 | 100% |
| *Alternaria infectoria* | 1 | AR | Feb2022 | 99.64% |
| *Aspergillus amstelodami* | 1 | AR | Feb2022 | 95.87% |
| *Alternaria alternata* | 1 | BH | Feb2022 | 99.45% |
| *Cephalotrichum gorgonifer* | 1 | BH | Feb2022 | 99.82% |
| *Coprinopsis cinerea* | 1 | BH | Feb2022 | 99.23% |
| *Rhizomucor pusillus* | 1 | BH | Feb2022 | 99.04% |
| *Rhizopus microsporus* | 1 | BH | Feb2022 | 98.32% |
| *Talaromyces piceae* | 1 | BH | Feb2022 | 97.03% |
| *Alternaria alternata* | 1 | BR | Feb2022 | 100% |
| *Paecilomyces variotii* | 1 | BR | Feb2022 | 97.72% |
| *Talaromyces cecidicola* | 4 | BR | Feb2022 | 99.55% |
| *Rhizopus arrhizus* | 1 | BU | Feb2022 | 94.46% |
| *Lichtheimia ramosa* | 1 | GH | Feb2022 | 99.75% |
| *Rhizomucor pusillus* | 1 | GH | Feb2022 | 98.73% |
| *Talaromyces piceae* | 1 | GH | Feb2022 | 99.63% |
| *Talaromyces purpureogenus* | 1 | GH | Feb2022 | 97.87% |
| *Microascus cirrosus* | 1 | GR | Feb2022 | 99.50% |
| *Talaromyces piceae* | 1 | GR | Feb2022 | 99.69% |
| *Alternaria infectoria* | 1 | GU | Feb2022 | 99.64% |
| *Penicillium citrinum* | 1 | GU | Feb2022 | 99.80% |
| *Rhizopus arrhizus* | 1 | GU | Feb2022 | 94.68% |
| *Alternaria alternata* | 4 | AH | May2022 | 99.67% |
| *Alternaria infectoria* | 2 | AH | May2022 | 99.82% |
| *Alternaria alternata* | 2 | AR | May2022 | 99.81% |
| *Alternaria infectoria* | 6 | AR | May2022 | 99.64% |
| *Alternaria infectoria* | 5 | AU | May2022 | 99.74% |
| *Alternaria alternata* | 2 | BH | May2022 | 99.91% |
| *Alternaria alternata, Alternaria alstroemeriae* | 1 | BH | May2022 | 98.87% |
| *Coprinellus radians* | 1 | BH | May2022 | 98.6% |
| *Coprinopsis cinerea* | 3 | BH | May2022 | 99.68% |
| *Rhizomucor pusillus* | 1 | BH | May2022 | 100% |
| *Talaromyces cecidicola* | 2 | BH | May2022 | 99.21% |
| *Alternaria alternata* | 10 | BR | May2022 | 99.49% |
| *Alternaria alternata, Alternaria tenuissima* | 1 | BR | May2022 | 99.44%, 99.43% |
| *Alternaria infectoria* | 4 | BR | May2022 | 99.55% |
| *Alternaria tenuissima, Alternaria alternata* | 1 | BR | May2022 | 99.26% |
| *Coprinopsis cinerea* | 1 | BR | May2022 | 99.24% |
| *Fusarium proliferatum* | 2 | BR | May2022 | 99.6% |
| *Phlebiopsis crassa* | 1 | BR | May2022 | 98.24% |
| *Talaromyces cecidicola* | 2 | BR | May2022 | 99.38% |
| *Talaromyces piceae* | 2 | BR | May2022 | 99.86% |
| *Talaromyces pinophilus* | 1 | BR | May2022 | 99.81% |
| *Alternaria infectoria* | 1 | BU | May2022 | 99.46% |
| *Bjerkandera adusta* | 3 | BU | May2022 | 99.55% |
| *Hyphodermella rosae* | 1 | BU | May2022 | 99.49% |
| *Phanerochaete concrescens* | 1 | BU | May2022 | 98.84% |
| *Phlebiopsis crassa* | 1 | BU | May2022 | 98.86% |
| *Polyporus arcularius, Polyporus brumalis* | 1 | BU | May2022 | 96.31%, 96.30% |
| *Talaromyces cecidicola* | 3 | BU | May2022 | 99.41% |
| *Talaromyces cecidicola, Talaromyces dendriticus* | 1 | BU | May2022 | 98.94% |
| *Alternaria alternata* | 3 | GH | May2022 | 99.75% |
| *Coprinopsis cinerea* | 1 | GH | May2022 | 98.92% |
| *Talaromyces aculeatus, Talaromyces verruculosus* | 1 | GH | May2022 | 97.98%, 98.17% |
| *Alternaria alternata* | 10 | GR | May2022 | 99.87% |
| *Alternaria alternata, Alternaria tenuissima* | 1 | GR | May2022 | 99.81% |
| *Alternaria infectoria* | 5 | GR | May2022 | 99.64% |
| *Alternaria tenuissima* | 2 | GR | May2022 | 100% |
| *Alternaria tenuissima, Alternaria alternata* | 1 | GR | May2022 | 99.81% |
| *Alternaria alternata* | 1 | GU | May2022 | 99.44% |
| *Aspergillus lentulus, Aspergillus fischeri* | 1 | GU | May2022 | 99.64%, 99.46% |
| *Alternaria infectoria* | 33 | AH | July2022 | 99.41% |
| *Paecilomyces variotii* | 1 | AH | July2022 | 99.49% |
| *Alternaria alternata* | 2 | AR | July2022 | 99.81% |
| *Alternaria infectoria* | 46 | AR | July2022 | 99.41% |
| *Rhizopus arrhizus* | 1 | AR | July2022 | 99.66% |
| *Alternaria infectoria* | 38 | AU | July2022 | 99.37% |
| *Alternaria rosae* | 1 | AU | July2022 | 99.48% |
| *Penicillium citrinum* | 4 | AU | July2022 | 99,50% |
| *Rhizomucor miehei* | 1 | BH | July2022 | 99.83% |
| *Rhizopus microsporus* | 2 | BH | July2022 | 99.47% |
| *Alternaria alternata* | 1 | BR | July2022 | 100% |
| *Alternaria infectoria* | 1 | BR | July2022 | 99.11% |
| *Aspergillus brasiliensis, Aspergillus niger* | 1 | BR | July2022 | 99.82%, 99.64% |
| *Penicillium citrinum* | 1 | BR | July2022 | 99.81% |
| *Penicillium janthinellum* | 1 | BR | July2022 | 99.64% |
| *Penicillium ochrochloron* | 1 | BR | July2022 | 99.26% |
| *Talaromyces cecidicola* | 2 | BR | July2022 | 100% |
| *Talaromyces diversus* | 1 | BR | July2022 | 99.64% |
| *Talaromyces piceae* | 1 | BR | July2022 | 97.04% |
| *Talaromyces radicus, Talaromyces islandicus* | 1 | BR | July2022 | 97.97%, 96.56% |
| *Talaromyces verruculosus* | 1 | BR | July2022 | 99.63% |
| *Talaromyces verruculosus, Talaromyces oumae-annae* | 2 | BR | July2022 | 99.21%, 99.55% |
| *Talaromyces verruculosus, Talaromyces pinophilus* | 1 | BR | July2022 | 99.45%, 99.27% |
| *Alternaria infectoria* | 2 | BU | July2022 | 99.47% |
| *Penicillium adametzii* | 1 | BU | July2022 | 96.78% |
| *Rhizomucor miehei* | 1 | BU | July2022 | 90.34% |
| *Talaromyces cecidicola* | 2 | BU | July2022 | 99.74% |
| *Talaromyces ramulosus* | 2 | BU | July2022 | 99.47% |
| *Talaromyces verruculosus* | 1 | BU | July2022 | 99.27% |
| *Alternaria alternata* | 1 | GH | July2022 | 99.44% |
| *Alternaria infectoria* | 3 | GH | July2022 | 99.28% |
| *Aspergillus fumigatus* | 1 | GH | July2022 | 99.64% |
| *Paecilomyces variotii* | 1 | GH | July2022 | 99.65% |
| *Rhizopus* sp. | 1 | GH | July2022 |  |
| *Syncephalastrum racemosum* | 1 | GH | July2022 | 97.98% |
| *Talaromyces amestolkiae* | 2 | GH | July2022 | 99.45% |
| *Alternaria infectoria* | 1 | GR | July2022 | 99.12% |
| *Penicillium brasilianum* | 3 | GR | July2022 | 99.46% |
| *Penicillium madriti* | 1 | GR | July2022 | 99.45% |
| *Rhizopus arrhizus* | 1 | GR | July2022 | 99.66% |
| *Talaromyces amestolkiae* | 1 | GR | July2022 | 99.45% |
| *Talaromyces piceae* | 1 | GR | July2022 | 99.64% |
| *Talaromyces pinophilus* | 1 | GR | July2022 | 99.45% |
| *Talaromyces pinophilus, Talaromyces verruculosus, Talaromyces oumae-annae* | 2 | GR | July2022 | 96.05%, 99.55%, 99.45% |
| *Talaromyces purpureogenus* | 1 | GR | July2022 | 99.64% |
| *Talaromyces verruculosus* | 1 | GR | July2022 | 99.62% |
| *Talaromyces verruculosus, Talaromyces oumae-annae* | 6 | GR | July2022 | 99.54%, 99.33% |
| *Talaromyces verruculosus, Talaromyces pinophilus* | 2 | GR | July2022 | 99.63%, 99.36% |
| *Alternaria infectoria* | 1 | GU | July2022 | 99.12% |

**^a^** The counts for a species in the sampling point of the specific sampling are represented.

^b^ AH: Araba Hospital. AR: Araba Rural. AU: Araba Urban. BH: Bizkaia Hospital. BR: Bizkaia Rural. BU: Bizkaia Urban. GH: Gipuzkoa Hospital. GR: Gipuzkoa Rural. GU: Gipuzkoa Urban.

^c^ The identity percentage for each identification is indicated, when the counts were more than one the mean value is shown.
